# Oosporein in *Phlebia centrifuga*: Prediction of Key Enzymes by a Combination of Multi-Omics and Molecular Dynamics

**DOI:** 10.1021/acs.jafc.6c08619

**Published:** 2026-07-27

**Authors:** Niklas Broel, Franziska V. Wengner, Johanna V. Stein, Flavius Popa, Stefan Janssen, Cathrin Spröer, Boyke Bunk, Fengjiao Xin, Binglin Li, Martin Gand

**Affiliations:** † Institute of Food Chemistry and Food Biotechnology, 9175 Justus Liebig University Giessen, Heinrich-Buff-Ring 17, 35392 Giessen, Germany; ‡ Black Forest National Park, Schwarzwaldhochstrasse 2, 77889 Seebach, Germany; § Algorithmic Bioinformatics, Justus Liebig University Giessen, Ludwigsplatz 13 − 15, 35390 Giessen, Germany; ∥ Leibniz Institute DSMZ—German Collection of Microorganisms and Cell Cultures, Inhoffenstraße 7B, 38124 Braunschweig, Germany; ⊥ Laboratory of Biomanufacturing and Food Engineering, Institute of Food Science and Technology, Chinese Academy of Agricultural Sciences, 100193 Beijing, China; # Institute of Food Science Technology Nutrition and Health (Cangzhou), 061001 Cangzhou, China; ¶ College of Food Science and Engineering, Northwest University, 710069 Xi’an, China

**Keywords:** basidiomycota, oosporein, multiomics, colorant, molecular dynamics

## Abstract

The red bibenzoquinone oosporein, a promising biocontrol agent with potential to replace conventional pesticides in insect pest management in crops, was produced by the Basidiomycota *Phlebia centrifuga* P. Karst isolated from the Black Forest National Park. A submerged system was established, yielding up to 1.60 g L^–1^ oosporein in the culture supernatant upon supplementation with the key intermediate orsellinic acid, which strongly induced oosporein-specific biosynthetic genes. Using a multiomics approach, genes encoding enzymes for all necessary conversions were predicted, including a type I polyketide synthase, two monooxygenases, and a heme peroxidase. Enzymatic functions were investigated by extensive docking analyses and molecular dynamics simulations, ultimately leading to the prediction of the underlying biosynthetic pathway. In summary, a spore-free, high-yield, scalable production platform for oosporein was established, highlighting the potential of rare, protected fungal species as sources for valuable enzymes and bioactive secondary metabolites for efficient microbial biomanufacturing systems.

## Graphical abstract

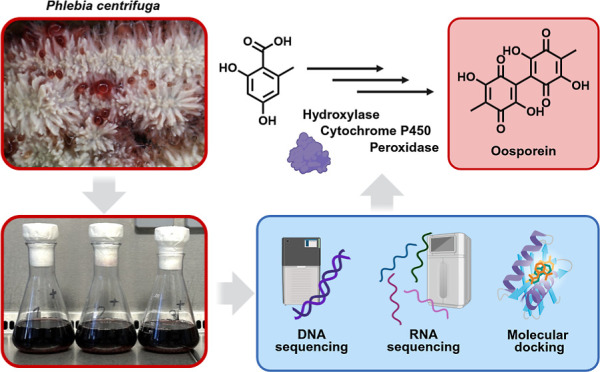

## Introduction

Oosporein, a symmetrical red 1,4-bibenzoquinone derivative, was first identified in the 1940s in the Ascomycota *Oospora colorans*,^1^ afterward the red colorant was isolated from other Ascomycota, for example, the insect-pathogenic fungi of the genus *Beauveria* e.g. *Beauveria bassiana* ^2,3^ or *Beauveria brongniartii*.^4^ Given the behavior of oosporein-producing organisms such as the insect-pathogenic *Beauveria* species, this secondary metabolite was identified as a promising candidate for biocontrol applications due to its broad spectrum of biological activities. Oosporein has been shown to exhibit remarkable antimicrobial properties against Gram-positive bacteria, in addition to antifungal activity against plant pathogens and insecticidal effects.^5–7^ These biological activities have been reported from a variety of entomopathogenic fungi, which are able to infect insects, including lepidopteran larvae, mosquitoes and beetles.^8–11^ While preparations of these Ascomycota species are already used as biocontrol agents, the exact role of oosporein has not yet been fully elucidated.^12^ It has been shown that oosporein does not directly kill insect larvae but rather plays various roles in insect pathogenesis. First, an immunosuppressive effect of oosporein is observed, promoting the infection with *Beauveria* sp.,^5,7,13^ second after the death of the larvae, it can facilitate the spread of the fungus through antibacterial effects.^6^ Which of these activities plays the pivotal role in entomopathogenesis remains controversial, but it can be concluded from all aforementioned studies that oosporein is necessary for an effective use of *Beauveria* sp. as a biocontrol agent.

As part of these investigations, the biosynthesis of oosporein in different *Beauveria* sp. as well as their regulatory cascades were examined. Feng et al.^7^ proposed a pathway for oosporein in *B. bassiana* and *B. brogniartii* centered on the key intermediate orsellinic acid, which is produced by a type I polyketide synthase (PKS). Subsequent oxidation as further conversion to oosporein, is proposed by the authors including several oxidoreductases, while they identified a Gal4-like Zn2Cys6 domain, containing a zinc finger protein as a key transcription factor of oosporein’s biosynthetic gene cluster (BGC).^7^ While further transcription factors operating in a cascade could be identified for *B. bassiana*,^6^ an analogous pathway to *B. bassiana* was identified for *Blackwellomyces cardinalis*, a further entomopathogenic Ascomycota.^11^

Although oosporein has been reported and investigated in numerous Ascomycota fungi, studies involving Basidiomycota remain limited. However, oosporein was identified from *Phlebia centrifuga* in 1965, formally known as *Phlebia mellea*. At that time, classification was based on morphological characteristics only.^14^ *P. centrifuga* P. Karst., is a white-rot causing corticoid fungus belonging to the Corticiaceae family within the Aphyllophorales order. It shows a circumboreal distribution but seems to be restricted to old-growth forests. *P. centrifuga* is characterized by its resupinate to effused-reflex fruiting body, which has a smooth to meruloid hymenophore and a pinkish-colored marginal zone. The order comprises many wood-decaying species that play an important ecological role in the decomposition of dead wood in boreal forests.^15^ The resupinate species was described in 1881, growing on *Picea abies* dead wood.^16^ *P. centrifuga* is considered as critically endangered in Germany (RL 1)^17^ and only few records in southern Germany are known. The species is known as an indicator species of unmanaged old-growth forests growing on large coniferous dead wood.^18,19^

The present study investigates the potential of *P. centrifuga*, a Basidiomycota fungus, as a high-yield submerged production system for oosporein. Oosporein itself might possess potential for utilization as a biocontrol agent, with the long-term goal of substituting conventional pesticides in agricultural contexts. While oosporein production has been documented in the *Phlebia* genus, deeper investigations into the possible yields and the biosynthetic pathway remain limited. Therefore, the genome of *P. centrifuga* was analyzed to obtain information about key enzymes and the biosynthetic pathway of oosporein in Basidiomycota. This work underscores the untapped potential of rare, fungal species for sustainable biotechnology. It illustrates their conservation value and establishes a basis for the biological production of bioactive secondary metabolites from fungal biodiversity.

## Materials and Methods

### Chemicals

Agar agar, boric acid (99.8%), d-glucose monohydrate, dimethyl sulfoxide-*d*_6_ (DMSO-*d*_6_, 99.9 atom % D), 2-propanol, magnesium sulfate monohydrate (99%), phosphoric acid (85%), sodium hydroxide (98%), triethylamine (99.5%) and yeast extract were purchased from Carl Roth (Karlsruhe, Germany). Acetonitrile (99.9%), ethanol (99.9%), formic acid (99.9%), malt extract, potassium dihydrogen phosphate (99.5%) and sodium acetate were obtained from Th. Geyer (Renningen, Germany). Glacial acetic acid, DMSO (99%), orsellinic acid monohydrate (98%), potato extract and sodium chloride (99.5%) were ordered from Fisher Scientific (Schwerte, Germany). Soy peptone and methanol (99.9%) were purchased from Merck (Darmstadt, Germany). Cellophane was acquired from Diagonal (Münster, Germany) and l-asparagine monohydrate from AppliChem (Darmstadt, Germany). Reference standard of oosporein (70%) was purchased from Cayman Chemical (Ann Arbor, MI, USA). Numbers in parentheses represent the minimum purity and the minimum deuteration level, respectively.

### Fungus Isolation and Maintenance

Fruiting bodies of *P. centrifuga* were collected in the Black Forest National Park inside the protected area formerly known as “Wilder See” from coniferous dead wood (*P. abies*/*Abies alba* dead wood; KR-M-0090335; 48.5690953 °N, 8.2377273 °E). A pure culture of *P. centrifuga* was created by spore shedding on a malt yeast peptone agar (7 g L^–1^ malt extract, 0.5 g L^–1^ yeast extract, 1 g L^–1^ soy peptone and 15 g L^–1^ agar agar) plate. Once the plate was approximately 80% covered with mycelium, again a 1 × 1 cm piece of overgrown agar was transferred to a malt extract agar (MEA; 30 g L^–1^ malt extract and 15 g L^–1^ agar agar) plate, where the strain was maintained. *P. centrifuga* was deposited at the German Collection of Microorganisms and Cell Cultures (DSMZ) under the identifier DSM 121802.

## Production, Isolation and Identification of Oosporein

Cultivation of *P. centrifuga* in potato dextrose medium (PD; 4 g L^–1^ potato extract, 22 g L^–1^ d-glucose monohydrate [autoclaved separately]) led to reliable formation of oosporein in the culture supernatant. Therefore, a 1 × 1 cm piece of overgrown agar was transferred into a 250 mL Erlenmeyer flask containing 100 mL PD medium. After homogenization via an Ultra-Turrax (Ika, Staufen, Germany) for 30 s, the cultures were kept in the dark at 24 °C on an orbital shaker (Orbitron, Infors, Bottmingen, Schweiz) at 150 rpm for 14 days. For the identification of the colorant in the culture supernatant, which turned from slightly yellow to bright red, the cultures were harvested and the mycelium was separated via centrifugation for 10 min at 4000*g* (Heraeus Megafuge 16 R, ThermoFisher, Waltham, MA, USA). Approximately 5 L of *P. centrifuga* culture supernatant were lyophilized (Christ Alpha 1–4 LSCplus, Martin Christ Gefriertrocknungsanlagen, Osterode am Harz, Germany) and the colorant was extracted three times with 400 mL methanol each. During the subsequent concentration step using a rotary evaporator (Rotavapor R-300, Büchi, Flawil, Switzerland), 291 mg of a bronze-colored precipitate formed. This precipitate was separated via filtration and further used for structure elucidation experiments. For the high-resolution electrospray ionization mass spectrometry (HR-ESI-MS) experiments performed with a Bruker Impact II quadrupole-time-of-flight instrument (Bruker, Billerica, MA, USA), the substance was dissolved in methanol with a concentration of 1 mg L^–1^. All nuclear magnetic resonance (NMR) spectra were recorded on a Bruker Avance NEO 700 MHz spectrometer (Bruker, USA) equipped with a 5 mm TCI H&F–C/N-D Prodigy CryoProbe at 303 K and 700 MHz for ^1^H and 176 MHz for ^13^C nuclei. All 1D spectra were referenced to the residual solvent signals of DMSO-*d*_6_ (δ_H_ 2.50 ppm, δ_C_ 39.5 ppm). A second set of NMR spectra was recorded after the addition of a 2 M excess of triethylamine (Et_3_N) to the sample.

### Quantification of Oosporein

To quantify oosporein from the culture supernatant, an adapted protocol from Seger et al. was used.^12^ 750 μL of culture supernatant was mixed with 750 μL BR5.5-MeOH buffer (Britton–Robinson buffer at pH 5.5 ^20^ mixed with methanol 3/7, v/v) and kept at −20 °C for 30 min. Afterward, any precipitate that may have formed was separated via centrifugation at 14,000*g* (Beckman Coulter Microfuge 22R, Brea, CA, USA). The samples were then subjected to reversed-phase high-performance liquid chromatography with diode array detection (RP-HPLC-DAD; Shimadzu Prominence LC-20AD equipped with an SPD M20DAD; Shimadzu, Kyoto, Japan). For all analyses a Hypersil Gold C18 column (150 mm × 4.6 mm, 5 μm, Thermo Fisher, USA) was used. The gradient program consisted of (A) dest. water with 0.9% formic acid and 0.1% acetic acid and (B) acetonitrile with 0.9% formic acid and 0.1% acetic acid and was set as follows: 1 min at 10% B, increasing the concentration of B to 60% in 14 min, afterward a steeper increase to 90% B in 2 min followed by final 2 min at 90% B. Bought oosporein, as analytical standard, was used for verification of the identified colorant as oosporein, while isolated oosporein was used for the external calibration.

## Increasing the Yield of Oosporein

To increase yields, the supplementation of orsellinic acid in submerged cultures was tested. Submerged cultures were prepared in PD medium, with one group of the cultures getting supplemented with orsellinic acid three times. This supplementation took place on days 7, 13, and 19 using 1.330 μL of an orsellinic acid stock solution (160 mM in DMSO), each time, reaching a final concentration of 2 mM orsellinic acid in the culture supernatant. Culture samples were taken on day 7, 8, 10, 12, 14, 16, 18, 20 and 22, the mycelium was separated via centrifugation at 14,000*g* (Beckman Coulter Microfuge 22R, USA) and discarded, while the supernatant was stored at −20 °C until HPLC-DAD analysis. The same procedure was also performed using standard nutrition solution (SNS) medium which was prepared according to Fraatz et al.^21^

### Generation of a Transcriptomic Database

To create a transcriptomic database, *P. centrifuga* was cultivated in both submerged and emersed culture form, using PD medium with and without orsellinic acid supplementation. For submerged cultivation, the previously described scheme was used. However, the scale was 40 mL of medium in a 100 mL flask and the cultivation period was only 12 days. Two groups of cultures were created: a control group in PD medium and a group in PD medium supplemented with 2 mM orsellinic acid (500 μL of a 160 mM stock solution in DMSO) on day 7. For sampling of both groups, three replicates, each consisting of four pooled flasks, were harvested on days 8, 10 and 12, giving a total of 18 samples. During harvesting, the mycelium and supernatant were first separated by centrifugation at 4000*g* (Heraeus Megafuge 16 R, ThermoFisher, USA). The mycelium was washed four times with a sterile 0.9% NaCl solution and then stored at −80 °C for RNA isolation. The supernatant was directly used for the quantification of oosporein as described above. For the emersed cultures of *P. centrifuga*, an overgrown 1 × 1 cm piece of agar was transferred either to a potato dextrose agar plate (PDA; 4 g L^–1^ potato extract, 22 g L^–1^ d-glucose monohydrate [autoclaved separately], 15 g L^–1^ agar agar) or a PDA plate with the initial supplementation of 2 mM orsellinic acid into the medium. A sterile cellophane film was placed on the surface of the agar plates to facilitate the separation of agar and mycelium. During harvesting on day 7, three plates were pooled. Supplemented and nonsupplemented medium was prepared in triplicates resulting in a total of six samples. Afterward, the mycelium was scraped from the agar plates, washed with sterile 0.9% NaCl and stored at −80 °C.

The mycelium of the submerged as well as the emersed cultivation was ground under liquid nitrogen and afterward the total RNA was isolated using the RNeasy Plant Mini Kit (Qiagen, Hilden, Germany) protocol for “Purification of total RNA from Plant cells and tissues and Filamentous Fungi” including the optional on-column DNA digestion using the RNase-Free DNase Set (Qiagen, Germany). Samples were then shipped to Novogene (Planegg, Germany) for mRNA library preparation and sequencing. This procedure included RNA quality control using Novogene’s capillary gel electrophoresis system, mRNA library preparation by poly­(A) enrichment, sequencing on a NovaSeq X Plus platform (6 Gb raw data per sample; Illumina, San Diego, CA, USA), and data quality control. The RNA-seq data are available under BioProject PRJNA1419734 (accession numbers SRX32139050–SRX32139073).

### Genome Isolation, Sequencing and Assembly

Genomic DNA from *P. centrifuga* was isolated using the Wizard HMW DNA Extraction Kit (Promega, Fitchburg, WI, USA). Eight aliquots were prepared according to the manufacturer’s instructions using the protocol “isolating HMW genomic DNA from plant tissue”. Subsequently, the samples were subjected to a 2-propanol/ethanol precipitation, in which 1/10 vol of sodium acetate buffer (3 M, pH 7.0) was added to the DNA solution, followed by 1 vol of 2-propanol and the samples were kept at −80 °C for 30 min. After centrifugation at 18,000*g* (Beckman Coulter Microfuge 22R, USA), the supernatant was discarded and the pellet was washed with 1 vol of 70% ethanol. After a second centrifugation step at 9000*g* the resulting pellet was air-dried for 15 min. Lastly, the pellet was dissolved in 15 μL rehydration solution provided in the kit and all aliquots were combined. Sequencing and long-read assembly of *P. centrifuga* genome were performed as described previously.^22^ The only deviations were the usage of the SMRTbell prep kit 3.0, the Sequel lle, while for assembly the “microbial genome analysis” in SMRTlink 13.1 was used. Complete genome sequencing data are available under BioProject PRJNA1419734 (accession number SRX32443382). For the phylogenetic analysis of *P. centrifuga*, a total of 305 nucleotide sequences representing six commonly used marker genes from 59 fungal species (File S1) were retrieved from NCBI. These comprised the internal transcribed spacer region of the 5.8S rRNA gene cluster (ITS_5.8S; *n* = 59), the nuclear ribosomal large subunit rRNA (nrLSU; *n* = 59), the nuclear small subunit rRNA (nuSSU; *n* = 49), the mitochondrial small subunit rRNA (mtSSU; *n* = 32), the translation elongation factor 1-α (EF-1α; *n* = 55), and the RNA polymerase II subunit gene (RPB2; *n* = 51). Each set of marker gene sequences was independently aligned via the multiple sequence alignment program MAFFT^23^ (v7.505, with parameters “--globalpair --maxiterate 1000”). The sequence alignments were turned into covariance models via cmbuild from the Infernal package^24^ (v1.1.4, with parameters “--noss”). As the bit scores of homology searches with covariance models depend on the length of the covariance model, which in turn depends on the initial marker gene sequence lengths, we scored each sequence against its corresponding covariance model via cmsearch (version 1.1.4, parameters “--tblout”) to obtain a reference threshold for discovery of novel members. Mean reference bit scores were EF-1α = 607.2 bits, ITS_5.8S = 359.9 bits, RPB2 = 812.3 bits, mtSSU = 455.5 bits, nrLSU = 1111.2 bits, nuSSU = 1344.2 bits. The same approach was used to search for hits of marker gene families in the genomic sequences of the studied *P. centrifuga* strain and additional 19 fungal species (File S1). The sequence of the top-scoring hit of each of the 20 genomes for each of the six marker gene models was added to the initial set of marker gene sequences if its bit score reached at least half the model-specific mean reference bit score. We found EF-1α = 20, ITS_5.8S = 16, RPB2 = 20, mtSSU = 4, nrLSU = 15, nuSSU = 17 novel hits, including hits for all six marker genes for the studied *P. centrifuga* strain. Via MAFFT, the marker gene sequences were aligned individually. The resulting six alignments were then concatenated to obtain one multiple sequence alignment in NEXUS format, consisting of 79 rows and 11,174 columns. Finally, IQTree^25^ (v1.6.12, with parameters “-nt AUTO -bb 5000′”) was used to construct the phylogenetic tree with partitioned maximum likelihood bootstrapping.

### Annotation, Expression Analysis and Selection of Genes of Interest

The intron/exon prediction, functional annotation, as well as the differential gene expression analysis (DGE) were performed as described in Broel et al. 2025.^22^ The assembled genome and a total of 24 mRNA libraries (18 from submerged and 6 from emersed cultures) were provided as raw data for the pipeline. In the differential gene expression analysis, the submerged cultures with supplementation (9 samples) were compared with those without (9 samples). During quality control of the triplicates, one of the triplicates from the nonsupplemented culture on day 7 (PCsPT13) was identified as an outlier in the expression heatmap and principal coordinate analysis (PCoA) and therefore was excluded (Figures S1 and S2), resulting in the final DGE comprehending 17 samples. The differentially expressed genes were sorted according to their upregulation in the supplemented group, and the 200 most upregulated genes were used to identify the genes of interest. These genes were further filtered for genes annotated as PKSs or as enzyme class (EC) 1 enzymes by the funannotate pipeline, while keeping all sequences with no annotated function. Sequences without annotation were subjected to a manual sequence annotation, using National Center for Biotechnology Information (NCBI) online Protein Basic Alignment Search Tool (BLASTp) service through a custom Python script to automatically perform sequence similarity searches. For each query sequence, the hit with the highest alignment score and available functional annotation was selected as the reference sequence. For some candidates, a homology search applying a Hidden Markov model was performed using phmmer^26^ at a cutoff at an *E*-value of ≤0.01 and a gap open penalty of 0.02 (v3.4, https://www.ebi.ac.uk/Tools/hmmer/search/phmmer). To further validate their potential functionality, protein structures were predicted and subsequently compared with homologous proteins, and potential enzymatic functions were inferred based on structural homology analysis.

### Docking Analysis

Protein structures were modeled using AlphaFold3.^27^ For enzymes containing cofactors, the cofactors were included during AlphaFold3 modeling, involving three types of cofactors (heme, flavin adenine dinucleotide [FAD]) and the reduced form of nicotinamide adenine dinucleotide ([NADH]). Each refined model was then subjected to molecular dynamics (MD) simulations. The microunit was solvated in a 90 × 90 × 90 Å^3^ cubic TIP3P water box using VMD v1.9.4,^28^ and all simulations employed the CHARMM45 force field.^29^ MD simulations were carried out with NAMD v3.0.1 under periodic boundary conditions.^30^ After 5000 steps of conjugate-gradient energy minimization, the systems were gradually heated to 313 K over 4 ns, followed by 200 ns production runs at a constant temperature and pressure. All other parameters were kept at their default values. MD trajectories were analyzed in VMD v1.9.4.^28^ Then, 200 representative snapshots were extracted at regular intervals from the 200 ns trajectory to construct a dynamic structural ensemble, which was subsequently used as the receptor library for docking to better account for protein flexibility. Molecular docking was performed on the MD-derived 200 representative structures of each enzyme. All docking calculations were performed using AutoDock Vina v.52ec525-mod,^31^ which employs the Broyden–Fletcher–Goldfarb–Shanno (BFGS) optimization scheme.^32^ Ligand and receptor structures were prepared in AutoDock Tools by converting PDB coordinates to PDBQT format, including assignment of atomic charges, atom types, and ligand rotatable bonds. A semiflexible protocol was adopted, with receptors treated as rigid and ligands fully flexible. The docking search space (grid boxes) was centered on the catalytic pocket of each enzyme.

## Results and Discussion

### Production, Isolation and Identification of Oosporein

When the fungus is cultivated submerged in PD medium, the culture supernatant shows a red coloration, as it can be observed at the edges or guttation drops of the wild fruiting bodies (Figure 1).

**Figure 1:**
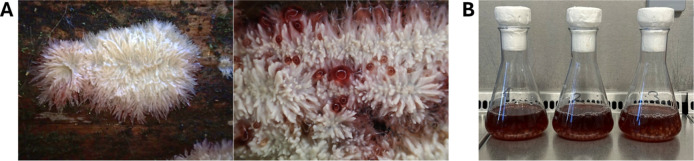
Pictures of the *P. centrifuga* strain used. (A) *P. centrifuga* fruiting body growing on coniferous dead wood in the Black Forest National Park (voucher KR-M-0090335) source of the strain (DSM 121802) used in this study. (B) Submerged cultures of *P. centrifuga* in PD medium.

The red colorant isolated from the supernatant of *P. centrifuga* PD cultures was identified via HRMS and NMR experiments. The isolate was subjected to HRMS (ESI-MS Bruker Impact II) in ESI­(+) mode, resulting in *m*/*z* values of 307.0446 [M + H]^+^ and 329.0267 [M + Na]^+^ (Figure S3), giving an exact mass of 306.0373 Da from which the molecular formula of oosporein C_14_H_10_O_8_ (Δ = −0.87 ppm) was calculated. ^1^H NMR spectra showed one signal at δ = 1.81 ppm (s, 6H) corresponding to methyl protons of C7/C7′ (Figure S4). ^13^C NMR spectra of oosporein in DMSO-*d*_6_ showed only three signals corresponding to six of oosporein’s 14 carbon atoms δ = 7.6, 107.4, and 112.8 ppm, corresponding to the two methyl carbons (C7/C7′) and the four quaternary carbons C1/C1′ and C4/C4′, respectively (Figure S5). The signals for the hydroxyl and carbonyl bearing carbons are most likely broadened by tautomerization as described previously for oosporein.^5,33^ Therefore, ^13^C NMR experiments were performed with the addition of 2 M excess of Et_3_N. These ^13^C NMR spectra gave signals at δ = 8.0 ppm (C7/C7′), 107.6 ppm (C1/C1′), 108.0 ppm (C4/C4′) and new signals at 171.4 ppm corresponding to the hydroxyl bearing carbon atoms C2, C5/C2′, C5′ and at 172.3 ppm assigned to the carbonyl carbon atoms C3, C6/C3′, C6′, respectively (Figure S6). Subsequent (^1^H/^13^C)-HMBC experiments showed ^2^ *J*_CH_ couplings between H7/H7′ and C4/C4′ and ^3^ *J*_CH_ couplings between H7/H7′ and C3/C3′ (Figure S7). Finally, the isolated substance was compared to a reference standard of oosporein analyzed via HPLC-DAD (Figure S8).

### Increase of Oosporein Yield

In line with the pathway for oosporein formation in *Beauveria* sp. postulated by Feng et al.,^7^ the supplementation of orsellinic acid resulted in a significant induction of oosporein production (Figure 2). While PD was confirmed to be the best medium for oosporein production, the same supplementation trials were conducted in SNS, to assess, whether oosporein formation could be induced similarly to PD medium. The supplementation of orsellinic acid to SNS thereby led to a significant increase in oosporein yield to a maximum of 0.37 g L^–1^, while the oosporein levels in nonsupplemented SNS were nearly nonexistent (∼0.01 g L^–1^). However, the supplementation in PD medium proved to be significantly more suitable, with about 4-fold higher yield of (1.60 ± 0.12) g L^–1^ at day 22 compared to SNS could be achieved by three-time supplementation of orsellinic acid.

**Figure 2:**
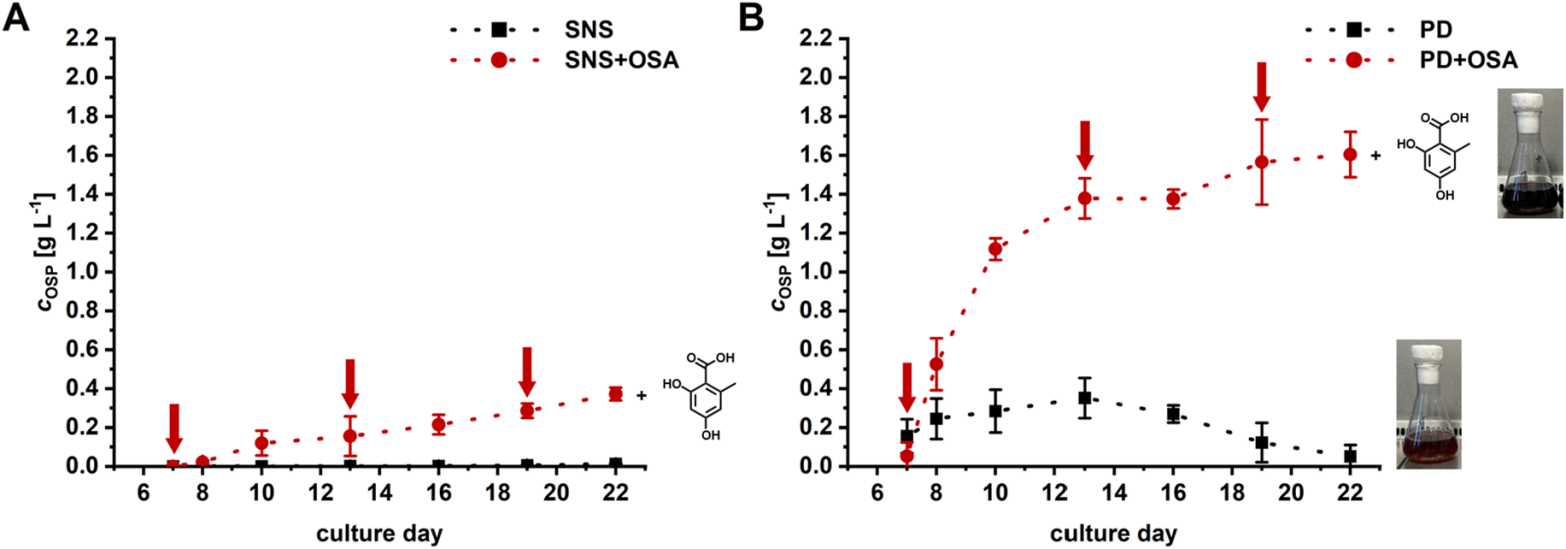
Induction of oosporein biosynthesis by orsellinic acid (OSA) in different media. Concentration of oosporein (*c*_OSP_) over time in the culture supernatants of *P. centrifuga* cultivated in (A) standard nutrition solution (SNS) and (B) potato dextrose (PD) medium, both with (red) and without (black) the addition of 2 mM OSA. Quantification was done via HPLC-DAD in biological triplicates whose standard deviations (SD) are represented by the error bars. Red arrows mark the days on which OSA was supplemented.

This yield is particularly remarkable when compared to literature values. For *B. bassiana*, for example, Fan et al.^6^ achieved a yield of 0.2 g L^–1^ through the overexpression of an oosporein-specific transcription factor, Lara-Juache et al.^34^ achieved 0.183 g L^–1^ in a biofilm reactor, and Amin et al.^13^ achieved 0.48 g L^–1^ oosporein in submerged cultivation using a glucose-rich medium. The overall highest yield from a submerged system to date was reported by Michelitsch et al.^35^ as 0.525 g L^–1^ in a stirred tank reactor. In terms of total oosporein concentration, the system described here achieves approximately 3-fold higher levels. These higher overall concentrations are especially advantageous for subsequent downstream processing. In general, the cultivation times with Ascomycota are lower, as demonstrated by the four-day cultivation period described by Michelitsch et al.^35^ However, it is crucial to emphasize that the Basidiomycota system is spore-free and in our cultivation system, yields of over 1 g L^–1^ can be achieved after 10 days of cultivation. In addition, it is noteworthy that there was a sharp induction after the addition of orsellinic acid, which resulted in a 10-fold increase in oosporein concentration in the culture medium from 0.05 to 0.53 g L^–1^ within 24 h of the initial supplementation of orsellinic acid on day 7. This increase continued until day 10, at which point a 20-fold increase was observed in comparison to day 7, reaching a concentration of already 1.12 g L^–1^ oosporein.

This suggests a clear induction of the oosporein-producing gene cluster and not just an additional conversion of orsellinic acid to oosporein, which is proven by the fact that only 0.31 g L^–1^ oosporein could be formed under a complete conversion of 2 mM orsellinic acid. Therefore, it can be assumed that the added orsellinic acid is already completely metabolized after 24 h and the induction of the oosporein pathway is responsible for further formation. It should also be noted that the latter two additions of orsellinic acid had little to no effect on the oosporein concentration, which only increased by a factor of ∼1.2 between days 13 and 22.

In summary, we were able to achieve the highest reported yield of oosporein in submerged cultivation, to the best of our knowledge. The decisive factor was the induction effect triggered by the addition of the key intermediate orsellinic acid, resulting in oosporein yields that render this Basidiomycota system economically attractive.

### Genome Assembly, Functional Annotation and Phylogenetics

The genome assembly of *P. centrifuga* resulted in 48 polished contigs, with an N_50_ contig length of 3.38 Mbp, totaling a genome size of 39.49 Mbp. The sequencing and assembly performed in this study were of high quality, especially when compared to the two reference genomes for *P. centrifuga* Phlcen1 ^36^ and ASM191385v2,^37^ which exhibit considerably higher contig numbers and markedly lower N_50_ contig lengths. Functional annotation via the funannotate pipeline resulted in 14,694 genes, representing the highest number of genes among these three genomes (Table 1).

**Table 1:** Key Genome Annotation Features of the Studied *P. centrifuga* Strain (DSM 121802) and the Two Reference Genomes ASM191385v2 and Phlcen1

	*P. centrifuga*	ASM191385v2 ^37^	Phlcen1 ^36^
genome size [Mbp]	39.5	34.6	34.9
no of contigs	48	1355	3667
N_50_ contig length [Mbp]	3.38	0.0729	n/a
no of genes	14,694	13,889	13,785

This high assembly quality is particularly important, as it ensures reliable gene prediction within the multiomics framework by reducing incomplete gene annotations that would otherwise occur for genes located on different contigs. The phylogenetic tree was calculated based on the sequences of the six genetic markers ITS_5.8S, nrLSU, nuSSU, mtSSU, EF-1α and RPB2. The studied organism clustered inside the phlebiod clade and showed high similarity to two *P. centrifuga* reference genomes Phlcen1 ^36^ and ASM191385v2 ^37^ confirming the studied organism as *P. centrifuga* (Figure S9).

### Prediction of the Oosporein Biosynthetic Pathway

For this high-yield oosporein-producing system, it was promising to delve deeper into the underlying biosynthetic machinery. To this end, a multiomics approach was implemented that, in addition to functional annotation, included DGE analysis focusing on genes differentially regulated in connection with oosporein formation. Both a submerged and an emersed cultivation trial were carried out in PD medium with and without orsellinic acid supplementation under determination of the oosporein concentration in the media (Figures 3A and S10).

**Figure 3:**
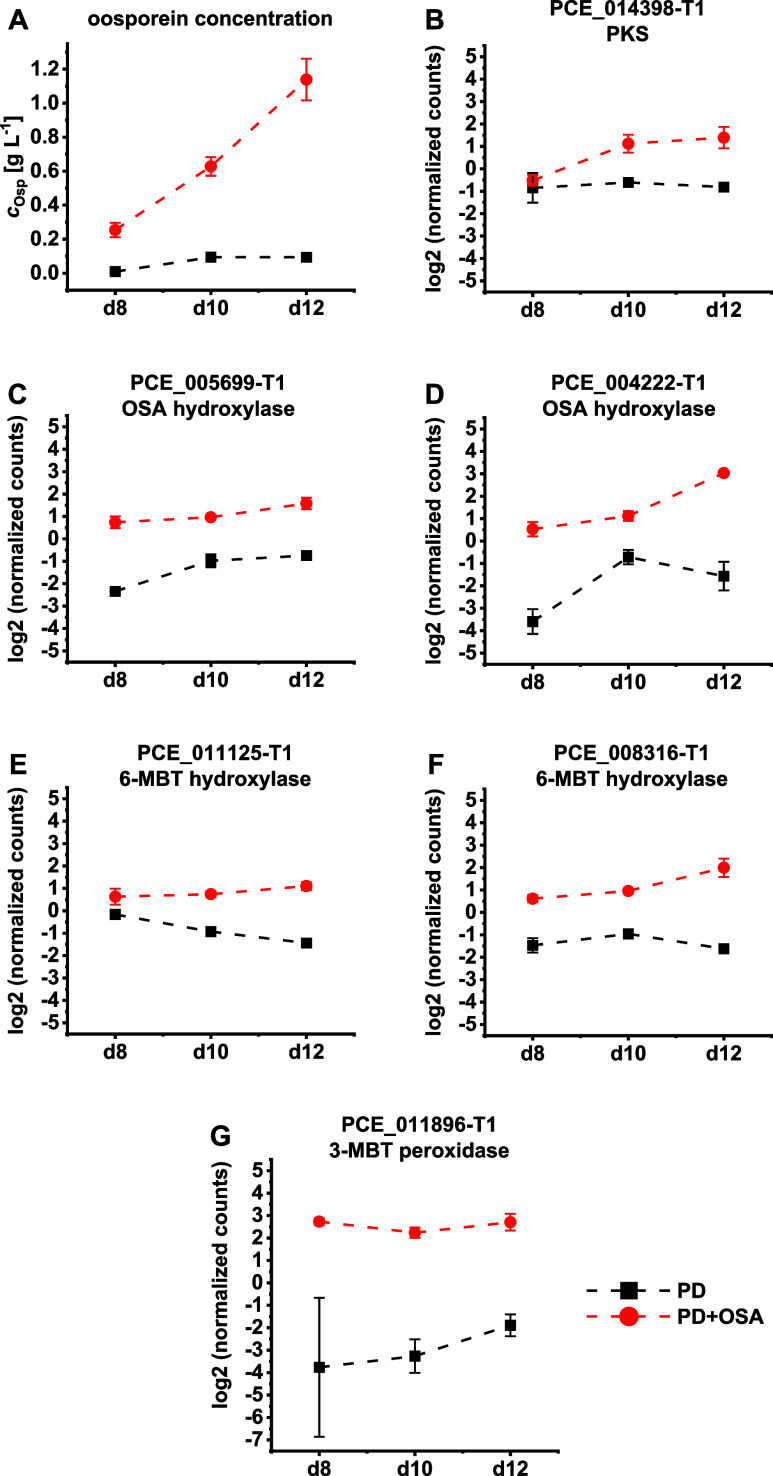
Transcript levels of putative oosporein biosynthetic genes in response to orsellinic acid (OSA) supplementation. (A) Concentration of oosporein (*c*_OSP_) in *P. centrifuga* supernatants at days 8, 10, and 12 determined via HPLC-DAD in PD medium (black) and PD medium supplemented with OSA (red), respectively. (B) Corresponding log2 normalized transcript counts for PCE_014398-T1, the putative OSA polyketide synthase (PKS). (C) Corresponding log2 normalized transcript counts for PCE_005699-T1, an OSA hydroxylase. (D) Corresponding log2 normalized transcript counts for PCE_004222-T1, an OSA hydroxylase (E) corresponding log2 normalized transcript counts for PCE_011125-T1, a putative 6-methyl-1,2,4-benzenetriol (6-MBT) hydroxylase (F) corresponding log2 normalized transcript counts for PCE_008316-T1, a putative 6-MBT hydroxylase and (G) corresponding log2 normalized transcript counts for PCE_011896-T1, a putative 3-methyl-1,2,4,5-benzenetetrol (3-MBT) heme peroxidase. The error bars represent the standard deviation (SD) of the biological triplicates.

Based on the pathway postulated by Feng et al. for *B. bassiana* pivoting around orsellinic acid as a key intermediate, the formation of oosporein in *P. centrifuga* was investigated. The biosynthesis of oosporein starts with a type I PKS, which synthesizes orsellinic acid from acetyl- and malonyl-CoA (Figure 4, step I).

**Figure 4:**
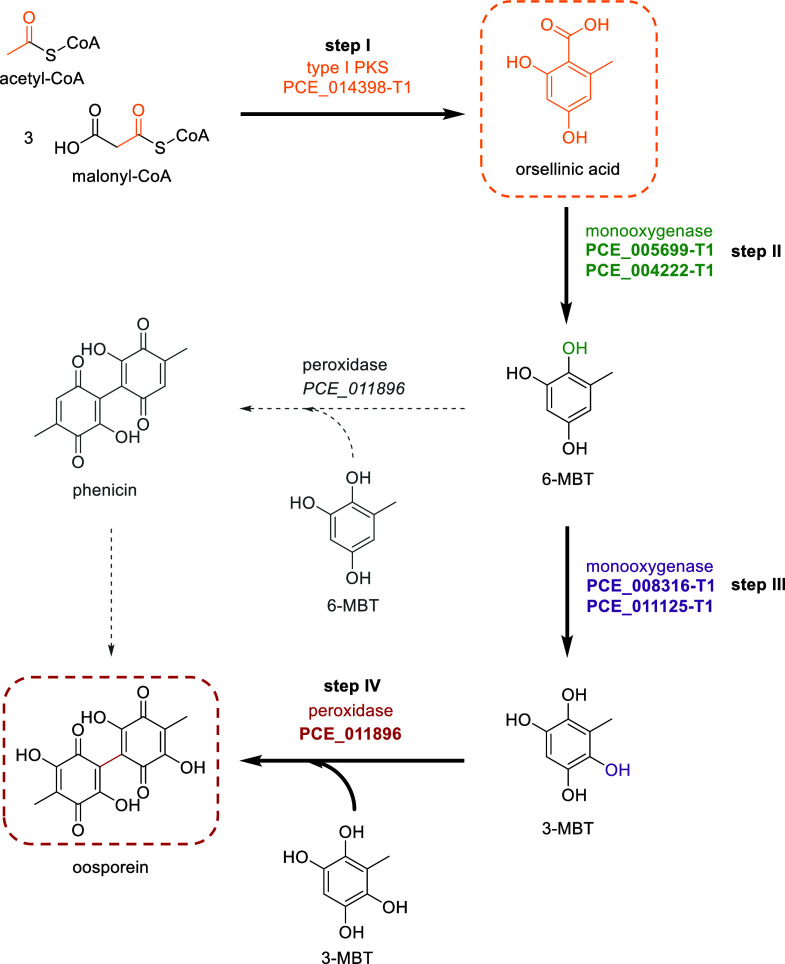
Proposed biosynthetic pathway of oosporein in *P. centrifuga*, including the key intermediate orsellinic acid as well as 6-methyl-1,2,4-benzenetriol (6-MBT) and 3-methyl-1,2,4,5-benzenetetrol (3-MBT). Functions of genes shown in bold were confirmed by molecular docking, whereas an alternative secondary route with phenicin as an intermediate was marked in gray. The enzymes required for steps I to IV, as well as the key atoms involved in these reactions, are shown in the colors orange, green, purple and red.

The candidate for this enzyme was the gene PCE_014398-T1, which was predicted via a protein BLAST search for the corresponding *B. bassiana* orsellinic acid PKS (OpS1) inside the annotated genes (File S2). When searching for proteins annotated as PKS within the highly differentially expressed genes of our transcriptomic approach, two further candidates, PCE_011117-T1 and PCE_014401-T1, were identified in addition to PCE_014398-T1. Protein BLAST analysis with three known orsellinic acid PKS from *Sparassis crispa*,^38^ *Armillaria mellea* ^39^ and *B. bassiana* revealed high identities especially for PCE_014398-T1 and the PKS from *S. crispa* (Table S1).

More detailed analysis of the sequences and domains with phmmer revealed that only PCE_014398-T1 possesses the SAT-KS-AT-DH-ACP-ACP-TE domain structure (SAT: starter domain; KS: β-ketoacyl synthase; AT: acetyltransferase; DH: dehydrogenase; ACP: acyl carrier protein; TE: thioesterase) similar to the reference PKS from the Basidiomycota *S. crispa* or *A. mellea* (Figure S11) or as it was proposed for OpS1 in *B. bassiana*.^7^ Expression levels for PCE_014398-T1 show a clear differential expression between the supplemented and nonsupplemented cultures, as well as an upward trend over the cultivation time in line with the observed oosporein concentrations (Figure 3A,B).

Subsequently in step II (Figure 4), orsellinic acid would be converted to 6-methyl-1,2,4-benzenetriol (6-MBT) via a decarboxylative hydroxylation. The best hit in the BLAST analysis based on the corresponding enzyme from *B. bassiana* (OpS4) was PCE_012468-T1 with an identity of 36.0% with a coverage of 66%. Since this enzyme was not among the 200 upregulated genes, it would be unlikely to be the right candidate and therefore was rejected for involvement in oosporein biosynthesis in *P. centrifuga*. In the next step, the 61 genes annotated as oxidoreductases among the 200 upregulated genes were manually subjected to a BLAST search to verify their annotated functionality and lower the number of candidates for a second BLAST step with selected references (File S3). Four candidates identified in this process were then subjected to the second BLAST analysis with four reference enzymes, which accept compounds structurally related to orsellinic acid as substrates in order to predict and validate the final candidates. Therefore, a 6-methylsalicylic acid decarboxylase from *S. crispa* (XP_027612085.1)^38^ and from *Aspergillus terreus* (AUZ97937.1),^40^ one salicylate hydroxylase from *Trametes pubescens* (OJT06981.1) and OpS4 (XP_008601501.1) from *B. bassiana* were used. PCE_005699-T1 and PCE_005698-T1 showed homology to the 6-methylsalicylic acid decarboxylase from *S. crispa*, PCE_004222-T1 for the salicylate hydroxylase from *T. pubescens* (Tables S2–S4). Notably, PCE_006102 exhibited low sequence identity with all reference sequences analyzed, suggesting an alternative function of this enzyme (Table S5), and was therefore excluded from further analysis. The remaining three candidates were then used for an in silico approach by docking analysis with the substrate orsellinic acid to prove their capability of orsellinic acid conversion to 6-MBT. Therefore, the required cofactors FAD and NADH were first docked into the protein structures created using AlphaFold3, and then a molecular docking analysis was performed with the substrate orsellinic acid. The affinity vs distance plots shows especially excellent results for the two enzymes PCE_005699-T1 and PCE_004222-T1, as in many frames docking of orsellinic acid was possible at a distance of less than 5 Å and an affinity of less than −4 kcal mol^–1^ (Figure S12). A more detailed analysis of the generated 3D structures revealed, on the one hand, the presence of the conserved **E-R-D** motif responsible for FAD binding^41^ within the active site of PCE_005699-T1, and, on the other hand, an alternative **D-R-D** motif for FAD binding in PCE_004222-T1 (Figure 5A,B).

**Figure 5:**
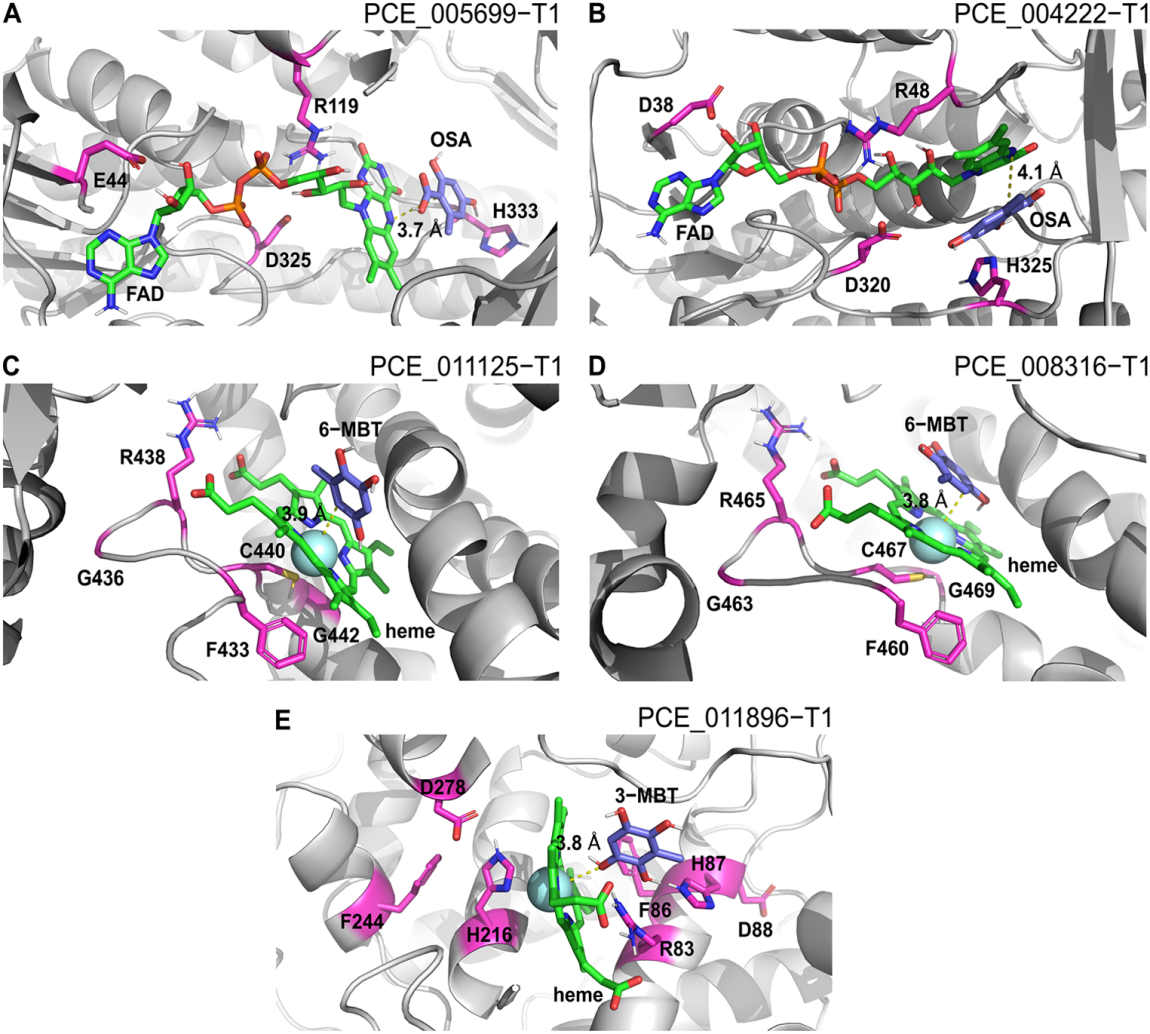
Molecular docking results for the proposed biosynthetic pathway genes of oosporein in *P. centrifuga*. Visualization of the docking results of orsellinic acid (OSA, blue) and the cofactors FAD (green) and NADH (not shown for clarity) for the putative decarboxylative hydroxylases (A) PCE_005699-T1 and (B) PCE_004222-T1. Visualization of the docking results of 6-methyl-1,2,4-benzenetriol (6-MBT, blue) and the cofactor heme (green, central Fe­(III) ion light blue) for the putative hydroxylases (C) PCE_011125-T1 and (D) PCE_008316-T1. Visualization of the docking results of 3-methyl-1,2,4,5-benzenetetrol (3-MBT, blue) and the cofactor heme (green, central Fe­(III) ion light blue) for (E) PCE_011896-T1. For each docking, the top-scoring pose was selected. Amino acid residues involved in ligand or substrate binding, as well as those with catalytic functions, are labeled and shown in magenta. The dashed yellow line marks the distance between the target atoms of cofactor and substrate.

Apart from that, these two enzymes possess a histidine residue that can interact with the ortho (PCE_004222-T1) or para hydroxy group (PCE_005699-T1) of orsellinic acid and thus support the hydroxylation reaction via general acid–base catalysis.^41^ In the case of PCE_005698-T1, the location of FAD in the protein did not appear to be ideal, and no complete motif for FAD binding could be identified, which raised concerns about the suitability of this sequence (Figure S13). In summary, the two candidates, PCE_005699-T1 and PCE_004222-T1, were selected as potential biocatalysts for the decarboxylative hydroxylation of orsellinic acid upon good results in the docking experiments, especially for PCE_005699-T1, and a clear upregulation of their transcript rates in correlation with the oosporein concentration (Figure 3A,C,D).

Continuing from 6-MBT, the main biosynthesis route for oosporein involves an additional hydroxylation step from 6-MBT to 3-methyl-1,2,4,5-benzenetetrol (3-MBT), followed by radical dimerization to oosporein (Figure 4, step III). Although direct dimerization of 6-MBT to phenicin, the dideoxy form of oosporein, with subsequent hydroxylation to oosporein is conceivable, especially this hydroxylation step has been ruled out for *B. bassiana* by Feng et al.^7^ Consequently, the focus was placed on the pathway traversing 3-MBT and the next step was the prediction of a 6-MBT hydroxylase. No hits were found via the BLAST search for OpS7 from *B. bassiana* inside the *P. centrifuga* genome (File S2) and many oxidative enzymes, which could possibly catalyze the hydroxylation from 6-MBT to 3-MBT, were found in the DGE. For that reason, a BLAST search with a 3-methylcatechol hydroxylase from *A. terreus* (AUZ97938.1) was performed. In this process, three genes PCE_011125-T1, PCE_008316-T1 and PCE_003489-T1, were identified, which were then used for docking experiments. The sequence PCE_011125-T1 showed a clustering of molecular dockings at slightly greater distances around 6 Å, with affinities around −4 kcal mol^–1^ (Figure S14). Favorable docking results were obtained for the remaining two candidates, with the dockings for PCE_008316-T1 clustering at particularly favorable affinities of less than −7 kcal mol^–1^ and those for PCE_003489-T1 clustering at particularly low distances of less than 3 Å. A more detailed investigation of the cytochrome P450 monooxygenase signature motifs **C-X-G** (**F-X-X-G-X-R-X-C-X-G**) for heme binding and **E-X-X-R** inside the K-helix was performed for these three candidates in order to verify them as functional P450 enzymes.^42^ For PCE_003489-T1, although the docking experiments showed small distances between the central Fe­(III) ion of heme and the substrate 6-MBT, no **C-X-G** motif could be identified, which raises questions about the catalytic capability of this sequence since especially the cysteine residue is considered invariant.^42^ Suitable heme binding pockets with complete **C-X-G** motifs as well as **E-X-X-R** motifs in the K-helix were identified for the two remaining candidates PCE_008316-T1 and PCE_011125-T1 (Table S6 and Figure 5C,D). Due to its more favorable affinities and distances in the docking plot compared to PCE_011125-T1, PCE_008316-T1 was identified as the most probable candidate for the conversion of 6-MBT to 3-MBT, while PCE_011125-T1 remains a plausible candidate. The final step IV (Figure 4) in the pathway is the dimerization of two 3-MBT molecules to form oosporein. The sequence PCE_011896-T1, annotated as a heme peroxidase, was selected for this conversion because this gene was strongly differentially expressed. Following a docking analysis was performed to verify the candidate and check whether this enzyme possesses a specificity toward 6-MBT or 3-MBT to gain information about the possible formation of phenicin. PCE_011896-T1 possesses a complete set of signature motives for the heme binding and heterolytic H_2_O_2_ cleavage site of class II heme peroxidases, namely the proximal **H-F-D** domain as well as the distal **R-X-X-F-H-D** motive (Figure 5E).^43,44^

Since the abstraction of a proton is generally possible at all hydroxyl groups of both 3-MBT and 6-MBT, dockings were performed with all positions, and the dockings of the best positions are shown (Figures 5E and S15). In general, the dockings show that conversion of both 3-MBT and 6-MBT is conceivable, therefore no specificity for either of the two biosynthetic routes can be observed based on the peroxidase, which might catalyze the dimerization of two building blocks to oosporein and phenicin, respectively (Figure S16). It should also be noted that peroxidases are generally less specific enzymes and therefore based on our data, the formation of phenicin in *P. centrifuga* cannot be ruled out completely.

It is noteworthy that the predicted genes in *P. centrifuga* are not organized into a BGC, in contrast to those identified in *Beauveria* spp., but are instead distributed across a broader genomic region. Oosporein biosynthesis in *P. centrifuga* therefore represents another example of two phenomena commonly observed in Basidiomycota. First, biosynthetic genes may be dispersed over substantially larger genomic regions compared to their compact organization in Ascomycota. Second, there appears to be a degree of functional redundancy among the genes involved, as indicated by the presence of multiple candidate genes for individual reaction steps in the work presented here.^45^ A well-documented example is the identification of five apparently redundant halogenases in *A. mellea*, all of which catalyze the same regioselective chlorination step in melleolide biosynthesis. Wick et al. (2016) showed that these genes are not located within the core melleolide biosynthetic gene cluster but are instead dispersed across large regions of the genome.^46^ Additional examples include six sesquiterpene synthases identified in *Coprinopsis cinerea*, only one of which is associated with a BGC,^47^ as well as a set of only weakly clustered genes involved in atromentin biosynthesis in *Paxillus involutus*.^48^

In summary, *P. centrifuga* was identified as a Basidiomycota species capable of producing oosporein at the g L^–1^ scale in a spore-free submerged cultivation system, establishing it as one of the most effective fungal sources of this compound reported to date. The high-quality genome sequenced and assembled in this study provided the essential foundation for integrated multiomics analyses, which in turn enabled the prediction of the underlying biosynthetic pathway. The investigations revealed orsellinic acid as the key intermediate, analogous to oosporein-producing Ascomycota and led to the discovery of promising biocatalysts, including a putative decarboxylative hydroxylase capable of converting orsellinic acid to the corresponding catechol. Much like other species isolated from the Black Forest National Park, such as *Cystostereum murrayi* ^49^ and *Laetiporus montanus*,^50^ *P. centrifuga* exemplifies that rare and protected fungal species can fulfill important ecological functions while simultaneously holding significant bioeconomic potential.

## Supplementary Material











## Notes

File S1: Reference sequences for the phylogenetic tree (XLSX)
File S2: BLASTp search results for *B. bassiana* genes (XLSX)
File S3: Selection of *P. centrifuga* genes and BLASTp comparison with selected references (xlsx)
File S4: PyMOL files containing the dockings for the genes of interest (ZIP)
PCoA plots and heatmaps of the transcriptomic data; HRMS, ^1^H-, ^13^C-, and HMBC-NMR spectra of oosporein; HPLC-DAD chromatograms of *P. centrifuga* culture supernatant and oosporein standard; phylogenetic tree of *P. centrifuga*; images of emersed cultivated *P. centrifuga* with and without orsellinic acid supplementation; BLASTp and phmmer analyses as well as distance/affinity plots of the selected genes; molecular docking results for PCE_005698-T1 and PCE_011896-T1; comparison of cytochrome P450 signature motifs for PCE_011125-T1 and PCE_008316-T1 (PDF)
