# Supplementary material for: Oosporein in Phlebia centrifuga: Prediction of Key Enzymes by a Combination of Multi-Omics and Molecular Dynamics
Source: J Agric Food Chem. 2026 Jul 27;74(30):23461–71. doi: 10.1021/acs.jafc.6c08619 (PMC13450407; doi:10.1021/acs.jafc.6c08619)
Supplement: Supplementary file 1 [file jf6c08619_si_004.pdf]

Supporting Information

Oosporein in *Phlebia centrifuga*: Prediction of Key Enzymes by a Combination of Multi-Omics and Molecular Dynamics

Niklas Broel[1], Franziska V. Wengner[1], Johanna V. Stein[1], Flavius Popa[2], Stefan Janssen[3], Cathrin Spröer[4], Boyke Bunk[4], Fengjiao Xin[5, 6], Binglin Li[7], Martin Gand[1,*]

[1] Institute of Food Chemistry and Food Biotechnology, Justus Liebig University Giessen, Heinrich-Buff-Ring 17, 35392 Giessen, Germany

[2] Black Forest National Park, Schwarzwaldhochstrasse 2, 77889 Seebach, Germany

[3] Algorithmic Bioinformatics, Justus Liebig University Giessen, Ludwigsplatz 13 – 15, 35390 Giessen, Germany

[4] Leibniz Institute DSMZ – German Collection of Microorganisms and Cell Cultures, Inhoffenstraße 7B, 38124 Braunschweig, Germany

[5] Laboratory of Biomanufacturing and Food Engineering, Institute of Food Science and Technology, Chinese Academy of Agricultural Sciences, 100193 Beijing, China

[6] Institute of Food Science Technology Nutrition and Health (Cangzhou), 061001 Cangzhou, China

[7] College of Food Science and Engineering, Northwest University, 710069 Xi'an, China

* Corresponding author: Martin Gand; E-Mail: Martin.Gand@lcb.chemie.uni-giessen.de, ORC-ID: 0000-0001-8211-691X

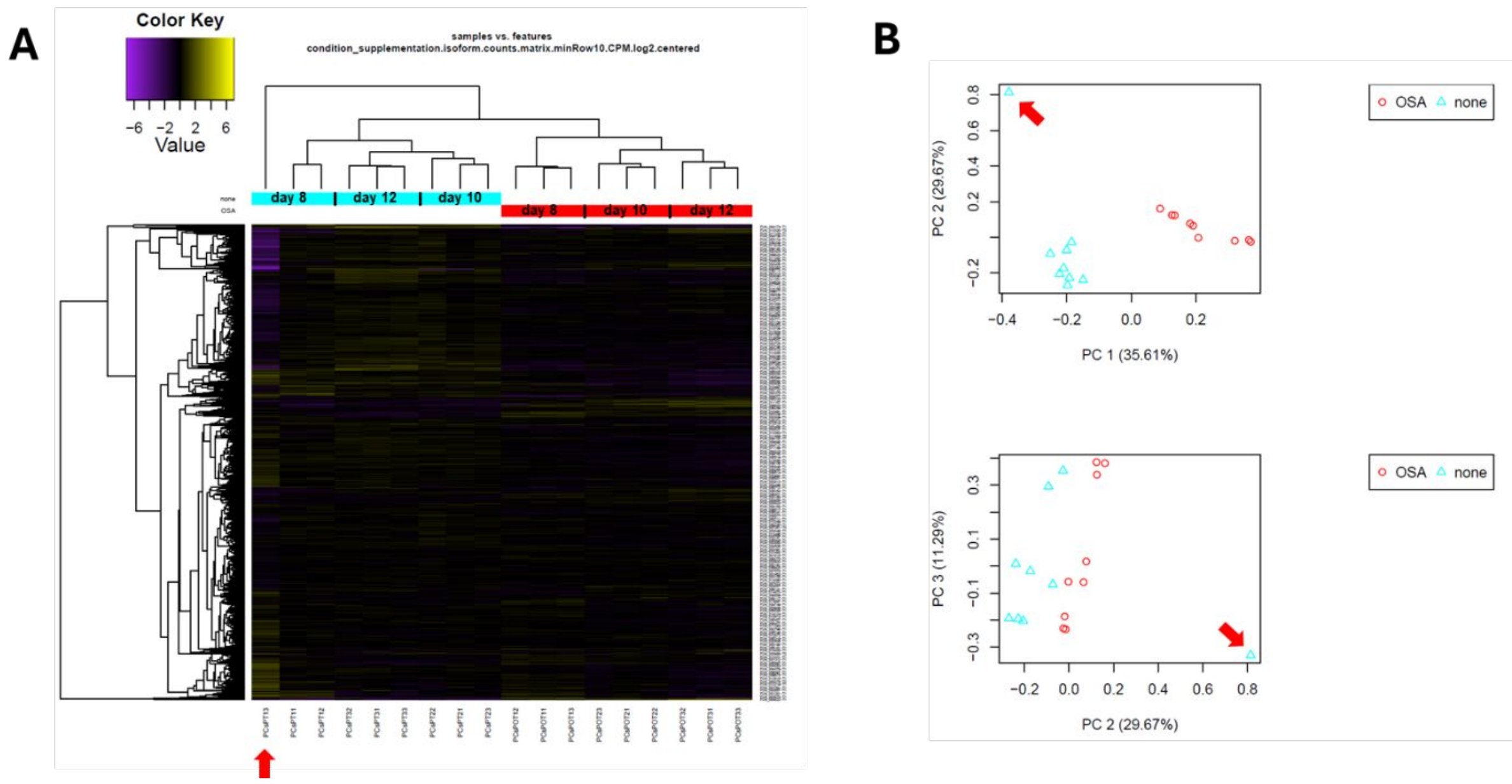

Figure S1: Heatmap (**A**) and principal coordinate analysis (PCoA) (**B**) for all 18 mRNA libraries with the condition non-supplemented (none, light blue) vs orsellinic acid (OSA, red). In (**A**) violet represents downregulated genes and yellow represents upregulated genes. Red arrows mark the identified outlier PCsPT13 in the PCoA as well as the expression heatmap.

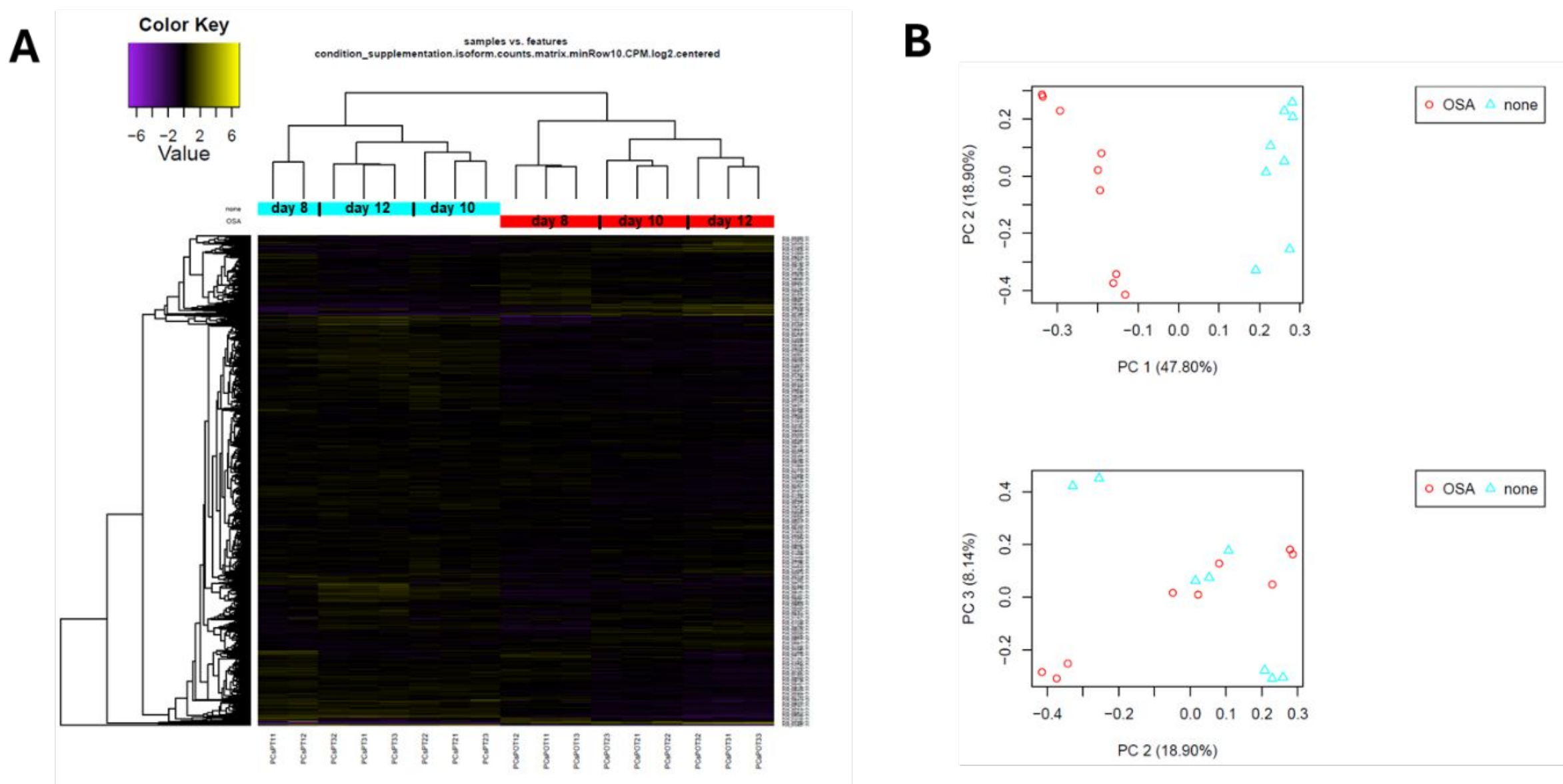

Figure S2: Heatmap (**A**) and principal coordinate analysis (PCoA) (**B**) for 17 mRNA libraries (without PCsPT13) with the condition non-supplemented (none, light blue) vs orsellinic acid (OSA, red). In (**A**) violet represents downregulated genes and yellow represents upregulated genes.

Generic Display Report

Analysis Info

Analysis Name	D:\Data\Data\2023\Broel\NB_000002.d	Acquisition Date	12/4/2023 1:38:19 PM
Method	Tune_pos_Standard.m	Operator	Analytik OC
Sample Name	HKP Peak 2	Instrument	impact II
Comment	MeOH<br>pos.		

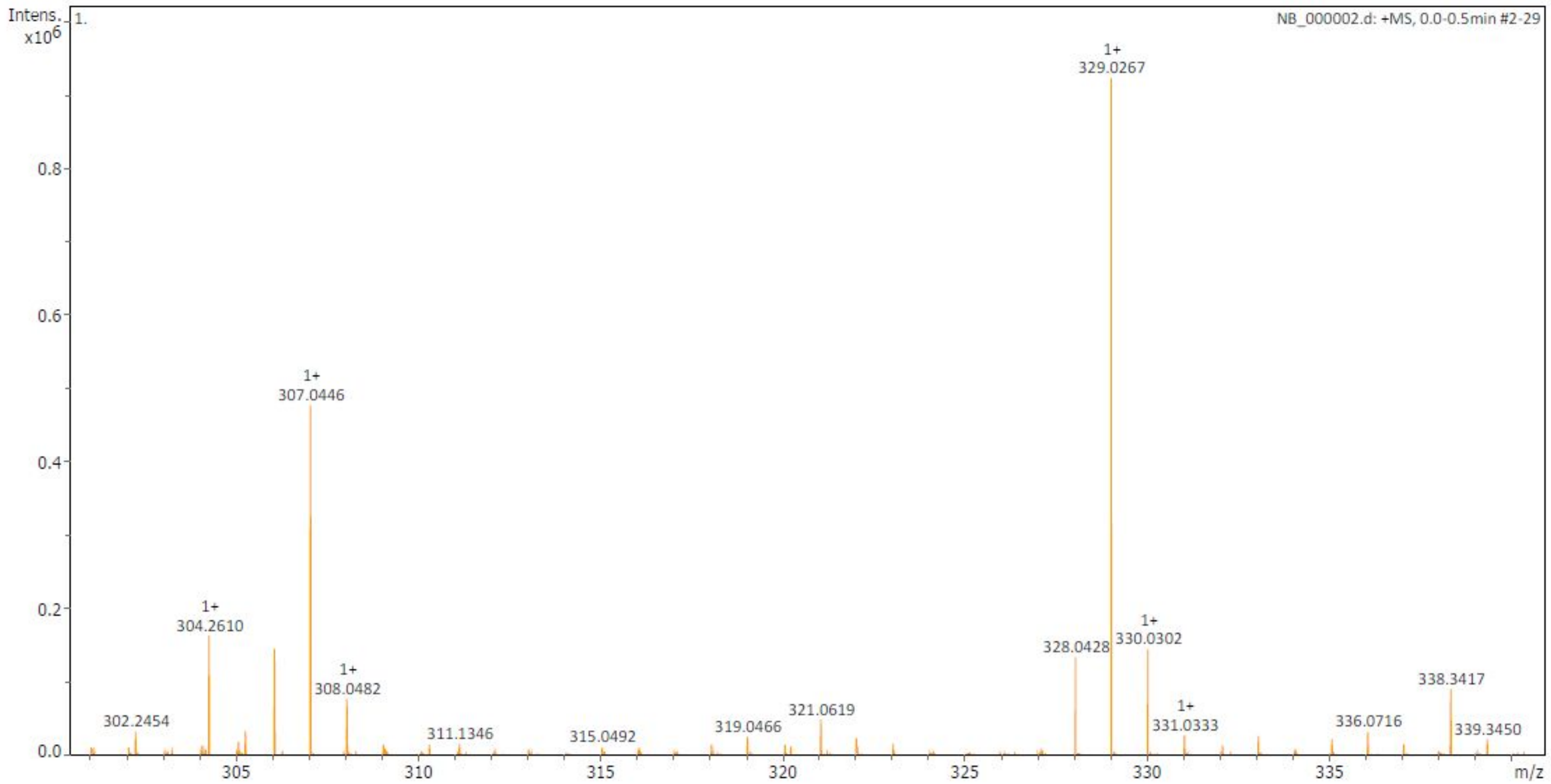

Figure S3: ESI(+)-HR-MS spectrum of isolated oosporein dissolved in MeOH.

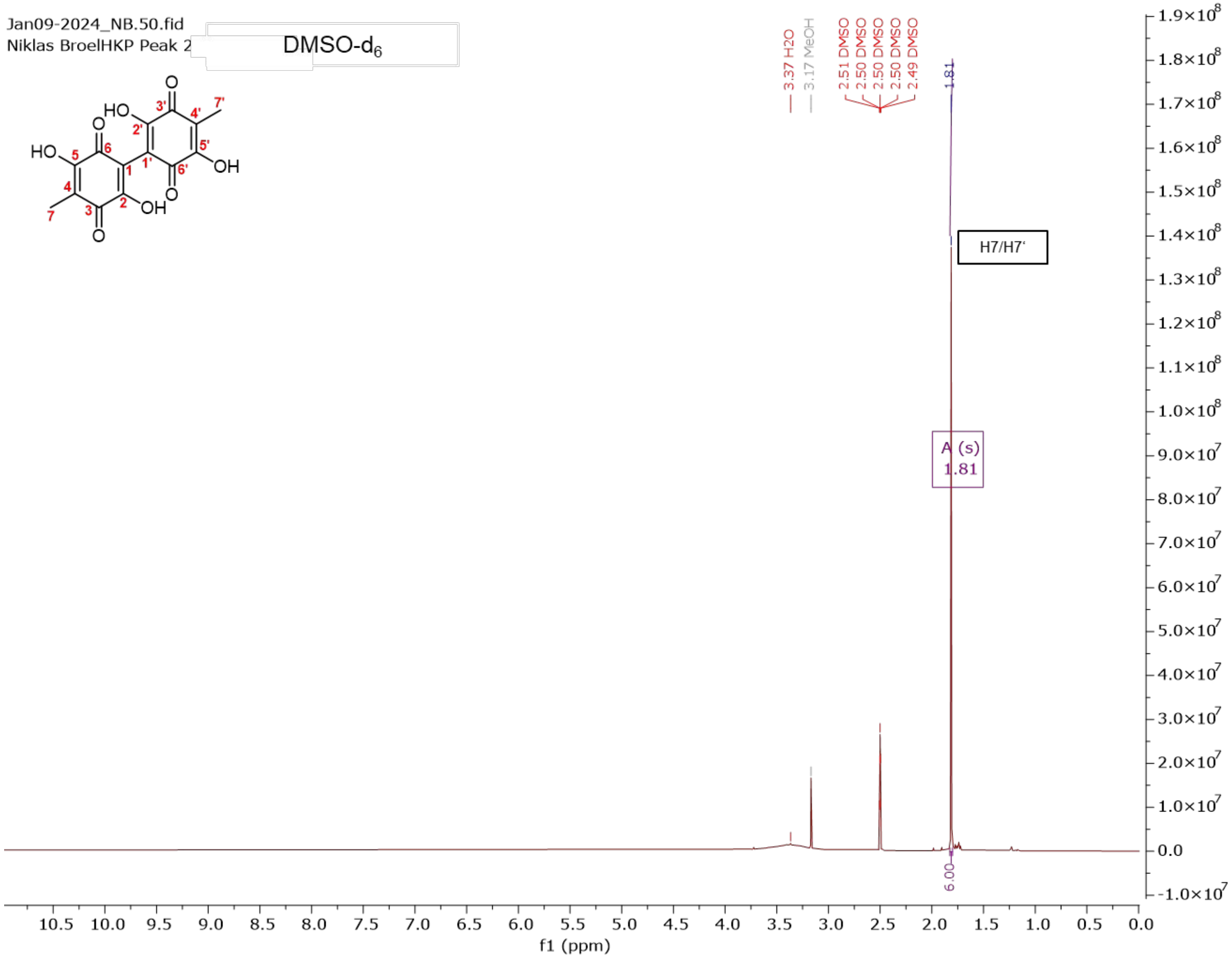

Parameter		Value
1	Title	Jan09-2024_NB.50.fid
2	Comment	Niklas BroelHKP Peak 2
3	Origin	Bruker BioSpin GmbH
4	Owner	nmrsu
5	Instrument	Avance Neo
6	Solvent	DMSO
7	Temperature	303.0
8	Pulse Sequence	zg30
9	Experiment	1D
10	Probe	Z168794_0004 (CPP1.1 TCI 700S3 H&F-C/N-D-05 Z XT)
11	Number of Scans	128
12	Receiver Gain	32.0
13	Relaxation Delay	1.0000
14	Pulse Width	7.9800
15	Acquisition Time	2.3593
16	Acquisition Date	2024-01-09T11:14:50
17	Modification Date	2024-01-09T11:14:51
18	Spectrometer Frequency	700.28
19	Spectral Width	13888.9
20	Lowest Frequency	-2625.6
21	Nucleus	1H
22	Acquired Size	32768
23	Spectral Size	131072
24	Digital Resolution	0.11

1H NMR (700 MHz, DMSO): δ 1.81 (s, 6H).

Figure S4: ^{1}H-NMR spectrum (700 MHz, 303 K, DMSO-d_6) of isolated oosporein.

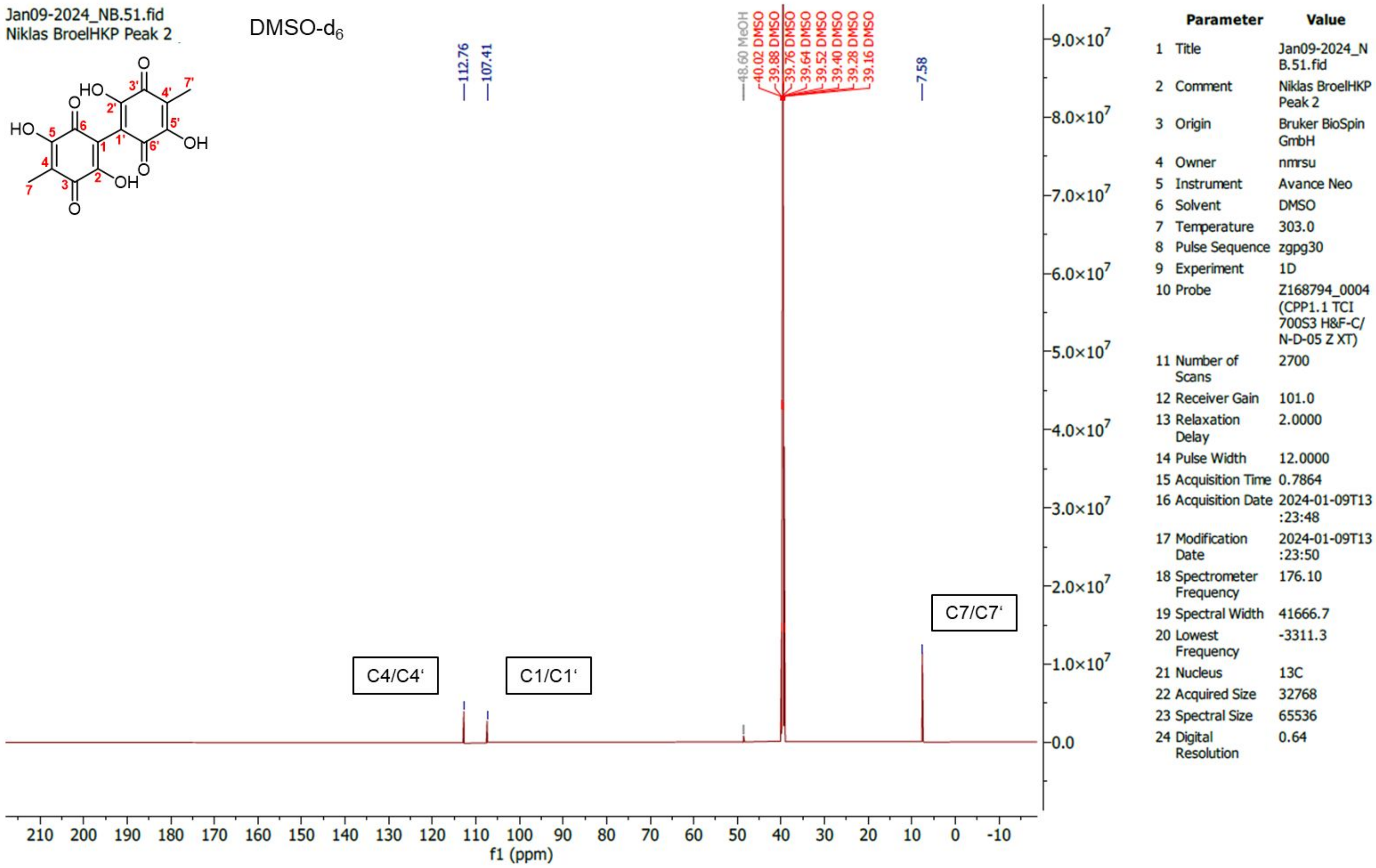

^{13}C NMR (176 MHz, DMSO) δ 112.76, 107.41, 48.60, 7.58.

Figure S5: ^{13}C-NMR spectrum (176 MHz, 303 K, DMSO-d_6) of isolated oosporein.

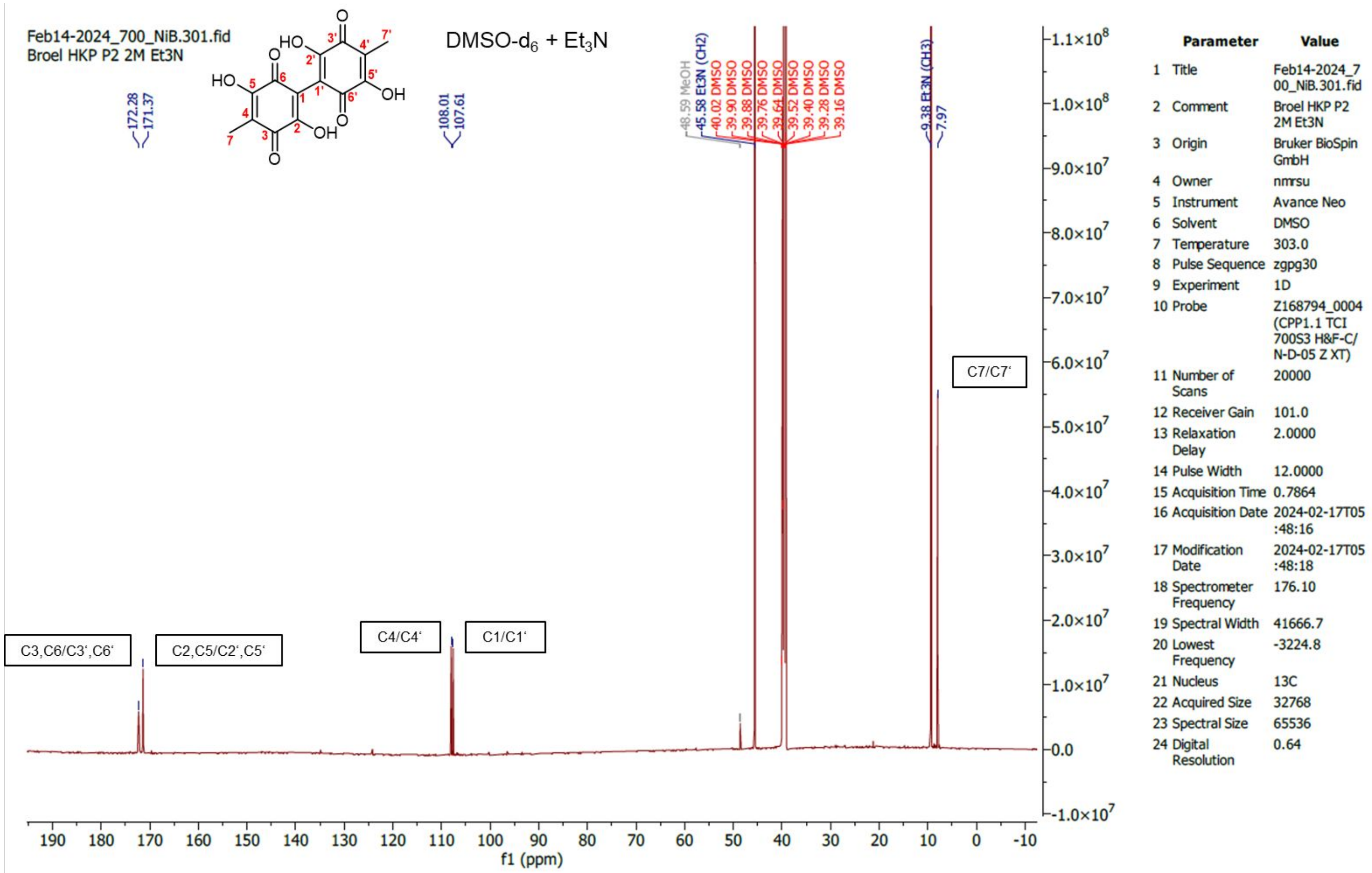

^{13}C NMR (176 MHz, DMSO) δ 172.28, 171.37, 108.01, 107.61, 48.59, 45.58, 9.38, 7.97.

Figure S6: ^{13}C-NMR spectrum (176 MHz, 303 K, DMSO-d_6 + Et_3N) of isolated oosporein.

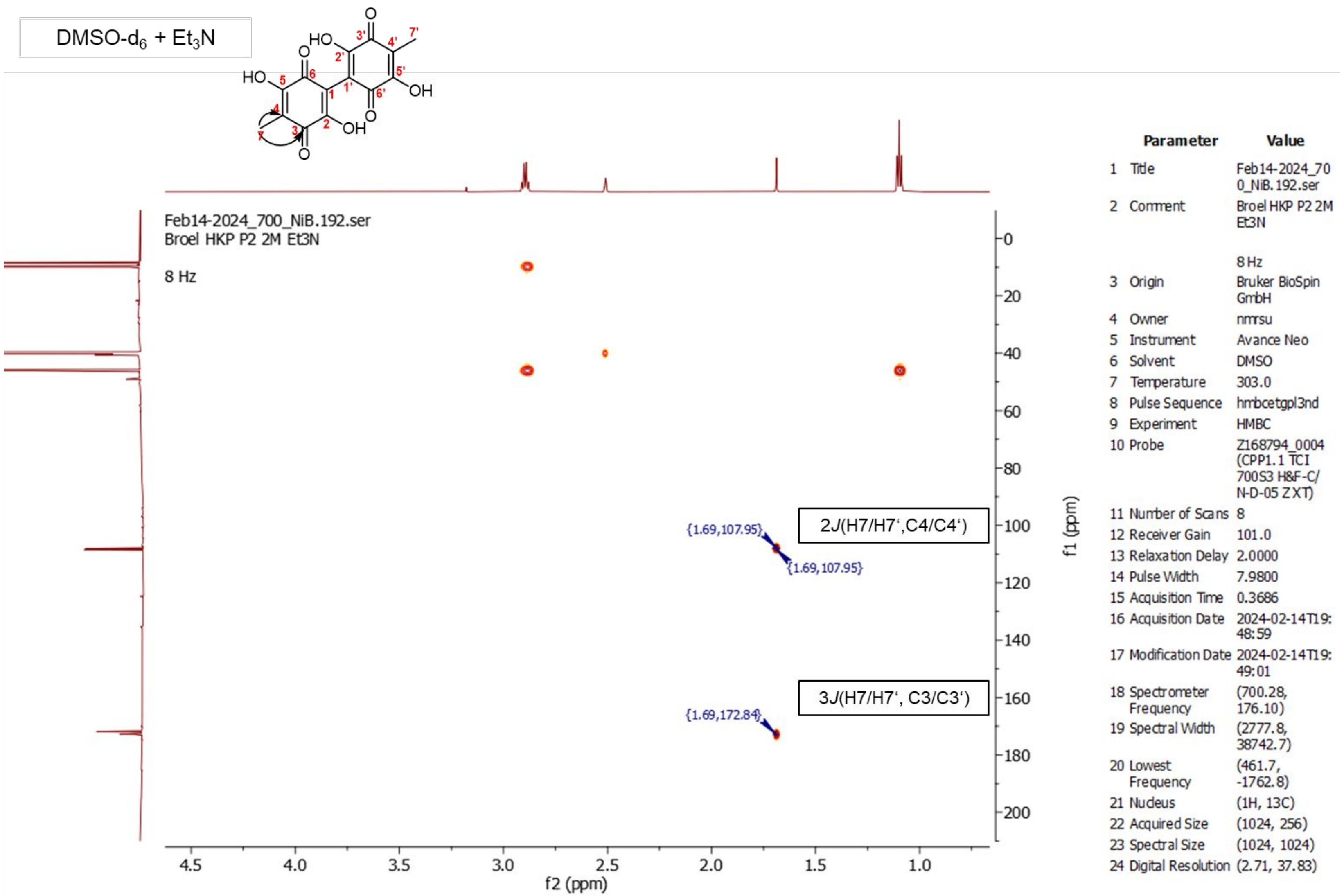

Figure S7: (^{1}H/^{13}C)-HMBC of isolated oosporein in DMSO-d_6 + Et_3N

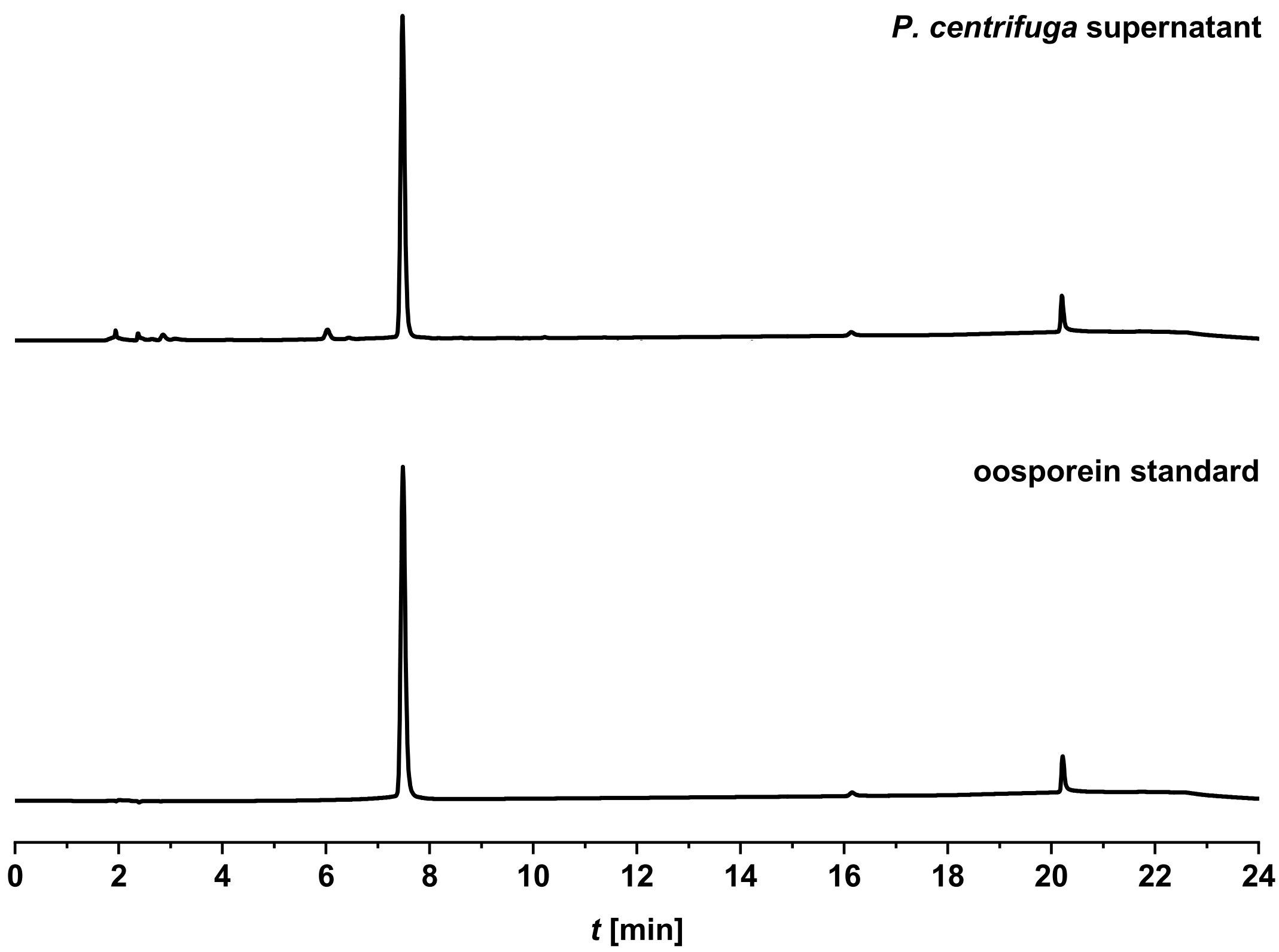

Figure S8: HPLC-DAD chromatograms of *P. centrifuga* culture supernatant (PD medium, day 18) and of an oosporein standard.

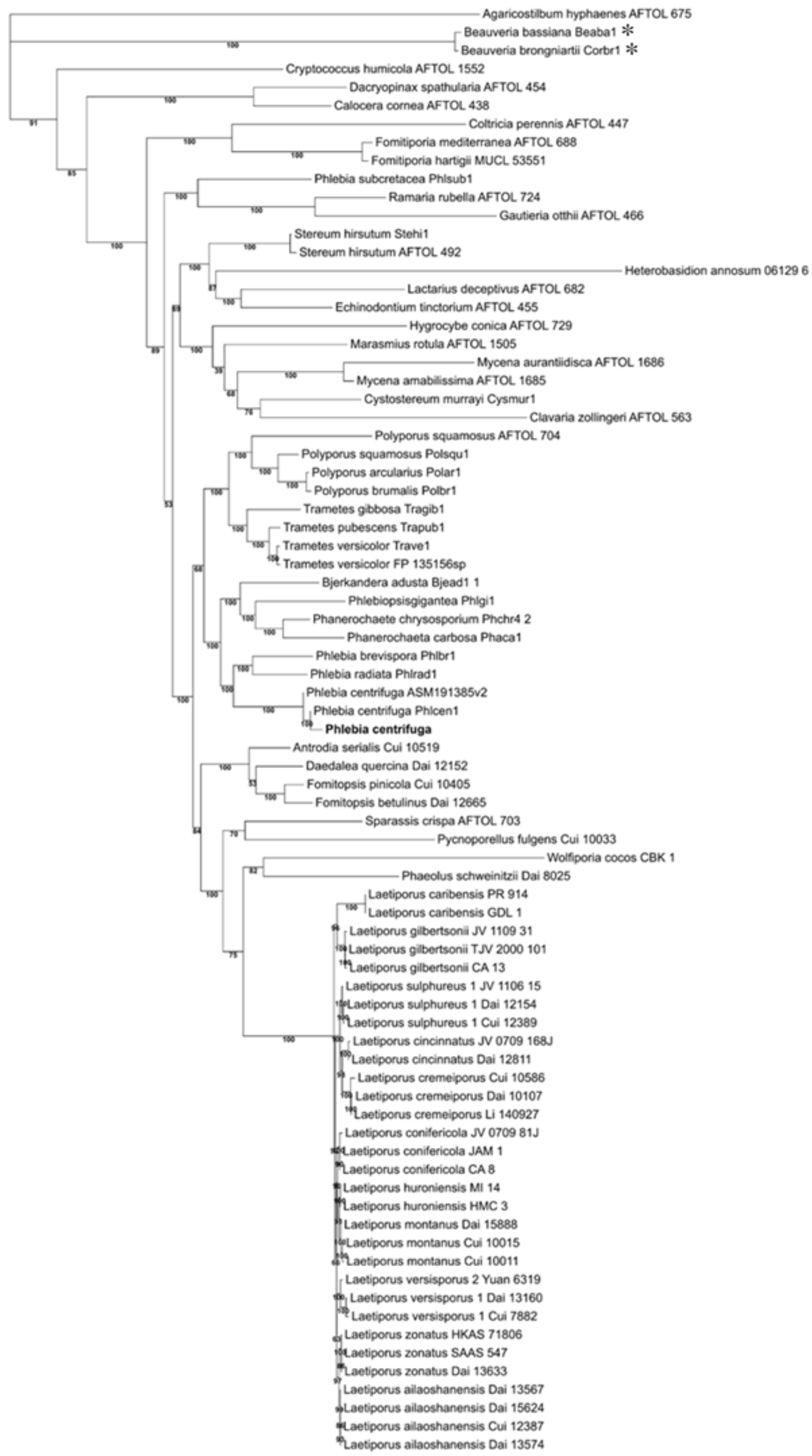

Figure S9: Phylogenetic tree based on EF-1α, ITS_5.8s, RPB2, mtSSU, nrLSU, nuSSU of 78 fungal genomes (File S2) as well as from the studied *P. centrifuga* (DSM 121802). The tree was reconstructed via IQTree [1] (v1.6.12, with parameters '-nt AUTO -bb 5000') with partitioned maximum likelihood bootstrapping and visualized via iToL. The asterisks mark the two Ascomycota.

[1] Minh, B. Q.; Schmidt, H. A.; Chernomor, O.; Schrempf, D.; Woodhams, M. D.; Haeseler, A. von; Lanfear, R. IQ-TREE 2: New Models and Efficient Methods for Phylogenetic Inference in the Genomic Era, Molecular biology and evolution. 2020, 37, pp. 1530–1534

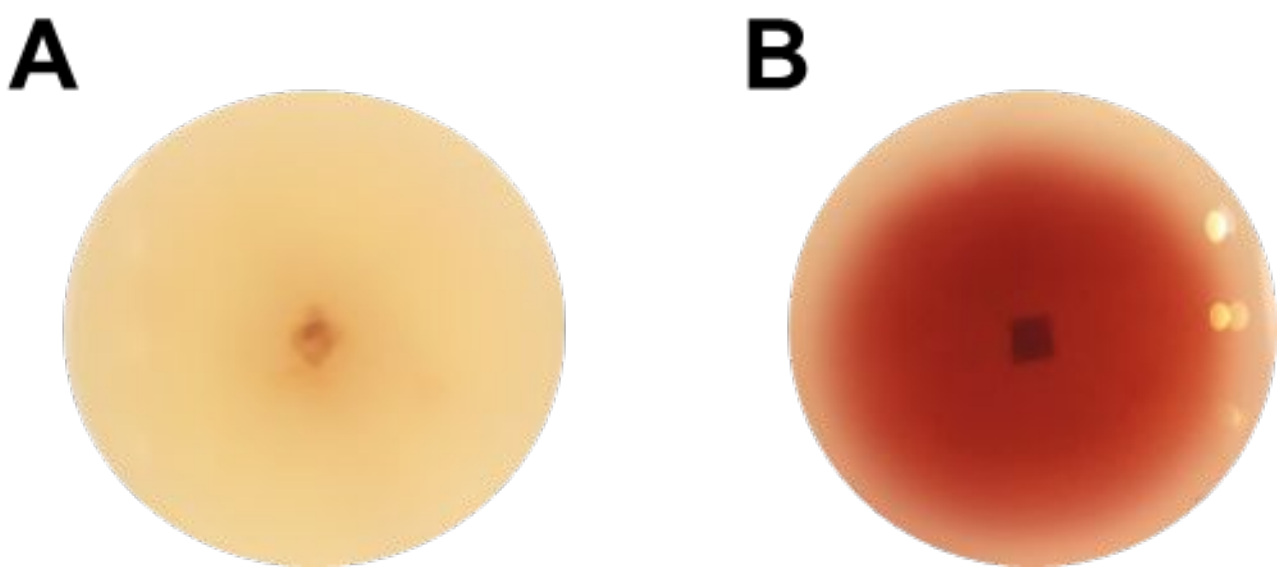

Figure S10: Emersed cultures of *P. centrifuga* on (**A**) PD and (**B**) PD with 2 mM orsellinic acid supplementation.

Table S1: BLAST analysis using **PCE_014398-T1** as the query and three reference orsellinic acid polyketide synthases (PKS) from *Sparassis crispa* (A0A401GFG2), *Armillaria mellea* (JQ801748.1) and *Beauveria bassiana* (XP_008601498.1) with the latter two being functionally confirmed as OSA PKSs *in vitro*. Ascomycota are marked with an asterisk.

Organism	Name	Coverage [%]	Identity [%]	Accession no.
Sparassis crispa		100	57.66	A0A401GFG2
Armillaria mellea	ArmB	99	42.27	JQ801748.1
Beauveria bassiana *	OpS1	99	35.95	XP_008601498.1

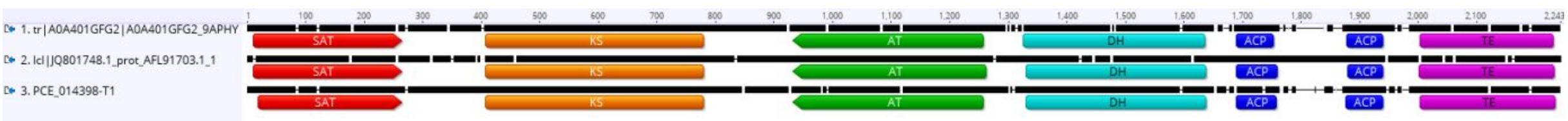

Figure S11: Domains assigned to **PCE_014398-T1** via phmmer search and comparison of **PCE_014398-T1** with orsellinic acid polyketide synthases (PKS) from *Sparassis crispa* (A0A401GFG2), *Armillaria mellea* (JQ801748.1) with the latter being functionally confirmed as OSA PKS *in vitro*.

Table S2: BLAST analysis using **PCE_004222-T1** as the query and four reference hydroxylases from *Sparassis crispa* (XP_027612085.1), *Aspergillus terreus* (AUZ97937.1), *Trametes pubescens* (OJT06981.1) and *B. bassiana* (XP_008601501.1). Ascomycota are marked with an asterisk.

Organism	Name	Coverage [%]	Identity [%]	Accession no.
Sparassis crispa	-	33	26.77	XP_027612085.1
Trametes pubescens	-	48	39.34	OJT06981.1
Aspergillus terreus *	AtA	65	23.05	AUZ97937.1
Beauveria bassiana *	OpS3	8	32.43	XP_008601501.1

Table S3: BLAST analysis using **PCE_005699-T1** as the query and four reference hydroxylases from *Sparassis crispa* (XP_027612085.1), *Aspergillus terreus* (AUZ97937.1), *Trametes pubescens* (OJT06981.1) and *B. bassiana* (XP_008601501.1). Ascomycota are marked with an asterisk.

Organism	Name	Coverage [%]	Identity [%]	Accession no.
Sparassis crispa	-	97	54.29	XP_027612085.1
Trametes pubescens	-	96	51.88	OJT06981.1
Aspergillus terreus *	AtA	96	35.21	AUZ97937.1
Beauveria bassiana *	OpS3	83	26.65	XP_008601501.1

Table S4: BLAST analysis using **PCE_005698-T1** as the query and four reference hydroxylases from *Sparassis crispa* (XP_027612085.1), *Aspergillus terreus* (AUZ97937.1), *Trametes pubescens* (OJT06981.1) and *B. bassiana* (XP_008601501.1). Ascomycota are marked with an asterisk.

Organism	Name	Coverage [%]	Identity [%]	Accession no.
Sparassis crispa	-	100	45.87	XP_027612085.1
Trametes pubescens	-	99	44.74	OJT06981.1
Aspergillus terreus *	AtA	84	30.67	AUZ97937.1
Beauveria bassiana *	OpS3	52	23.61	XP_008601501.1

Table S5: BLAST analysis using **PCE_006102-T1** as the query and four reference hydroxylases from *Sparassis crispa* (XP_027612085.1), *Aspergillus terreus* (AUZ97937.1), *Trametes pubescens* (OJT06981.1) and *B. bassiana* (XP_008601501.1). Ascomycota are marked with an asterisk.

Organism	Name	Coverage [%]	Identity [%]	Accession no.
Sparassis crispa	-	64	21.43	XP_027612085.1
Trametes pubescens	-	59	23.97	OJT06981.1
Aspergillus terreus *	AtA	90	23.12	AUZ97937.1
Beauveria bassiana *	OpS3	89	22.99	XP_008601501.1

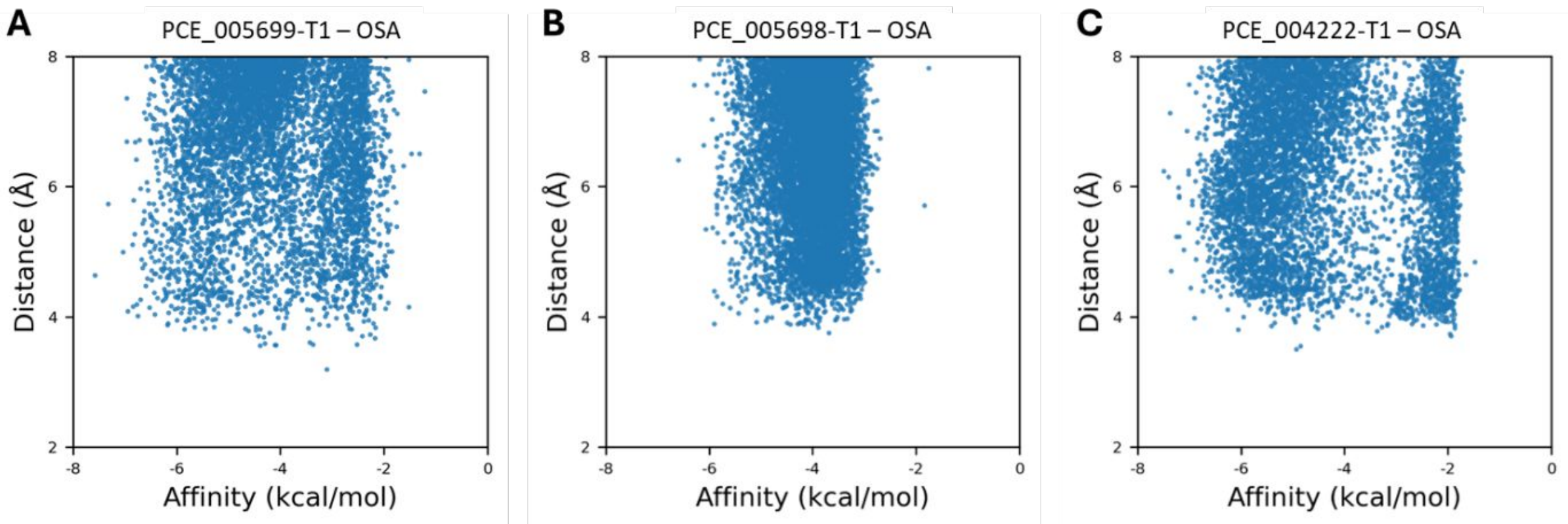

Figure S12: Distance/Affinity plots for the dockings of putative orsellinic acid (OSA) hydroxylase enzymes (**A**) **PCE_005699-T1**, (**B**) **PCE_005698-T1** and (**C**) **PCE_004222-T1**.

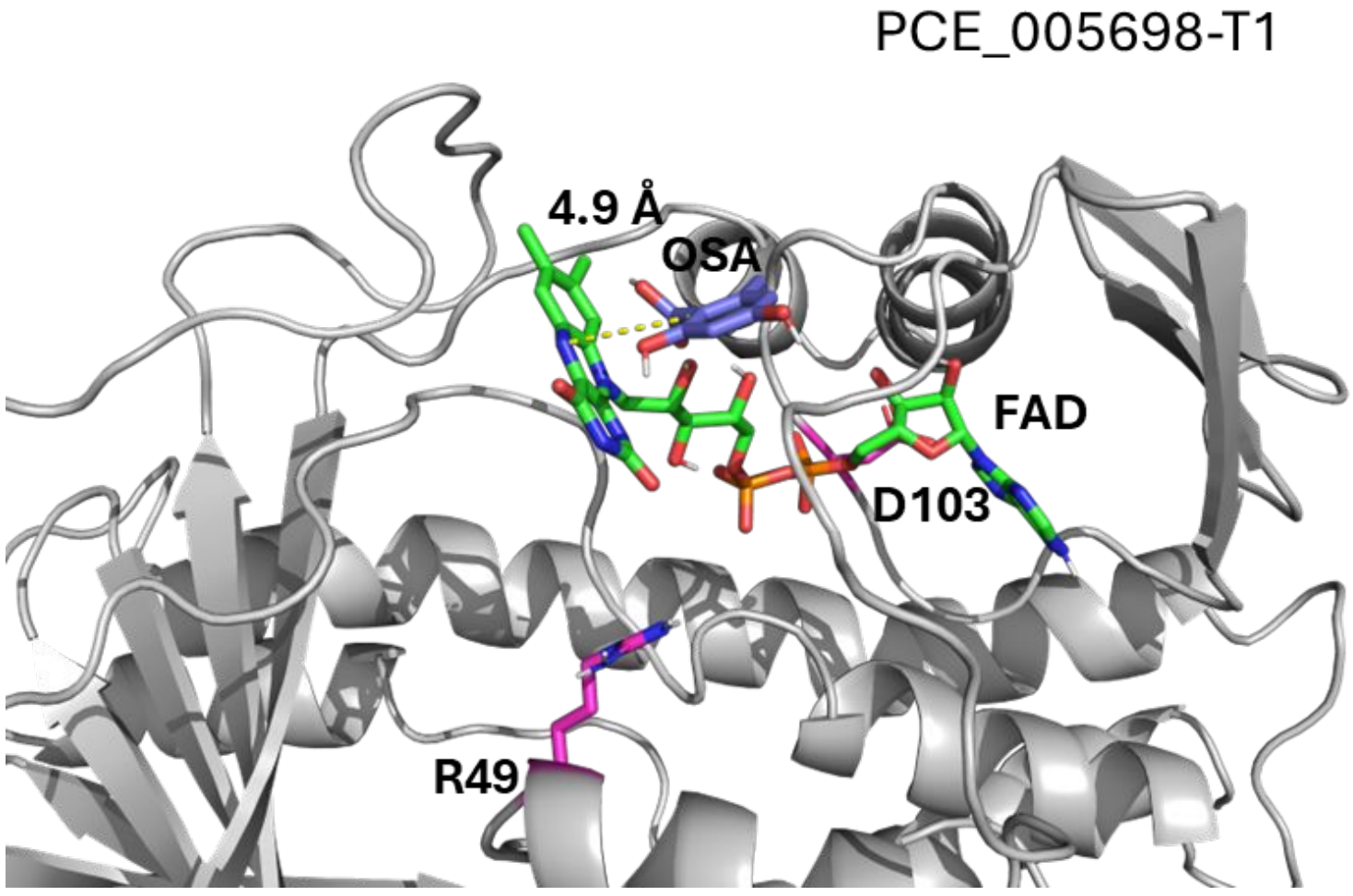

Figure S13: Visualization of the docking results of orsellinic acid (OSA, blue) and the cofactors FAD (green) and NADH (not shown for clarity) for the putative decarboxylative hydroxylase **PCE_005698-T1**. For the docking, the top-scoring pose was selected. Amino acid residues involved in ligand or substrate binding, as well as those with catalytic functions, are labeled and shown in magenta. The dashed yellow line marks the distance between the target atoms of cofactor and substrate.

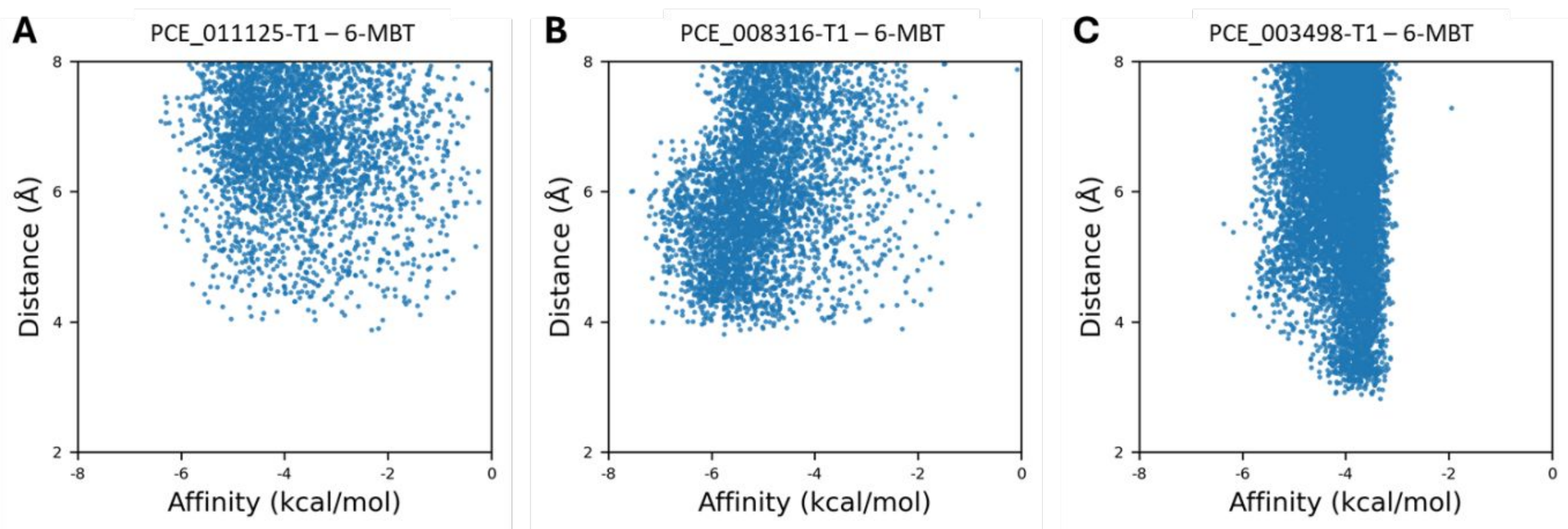

Figure S14: Distance/Affinity plots for the dockings of putative 6-MBT hydroxylase enzymes (**A**) **PCE_011125-T1**, (**B**) **PCE_008316-T1** and (**C**) **PCE_003489-T1**.

Table S6: Positions of the Cytochrome P450 signature motifs **C-X-G** and **E-X-X-R** identified in **PCE_011125-T1** and **PCE_008316-T1**.

	Heme binding pocket (C-X-G)									
Position	**1**	2	3	**4**	5	**6**	7	**8**	9	**10**
Motif	**F**	X	X	**G**	X	**R**	X	**C**	X	**G**
PCE_011125-T1	$\mathbf{F}_{433}$	G	Y	$\mathbf{G}_{436}$	R	$\mathbf{R}_{438}$	I	$\mathbf{C}_{440}$	P	$\mathbf{G}_{442}$
PCE_008316-T1	$\mathbf{F}_{460}$	G	F	$\mathbf{G}_{463}$	R	$\mathbf{R}_{465}$	V	$\mathbf{C}_{467}$	P	$\mathbf{G}_{469}$

	K-helix			
Position	**1**	2	3	**4**
Motif	**E**	X	X	**R**
PCE_011125-T1	$\mathbf{E}_{358}$	I	L	$\mathbf{R}_{361}$
PCE_008316-T1	$\mathbf{E}_{384}$	S	I	$\mathbf{R}_{387}$

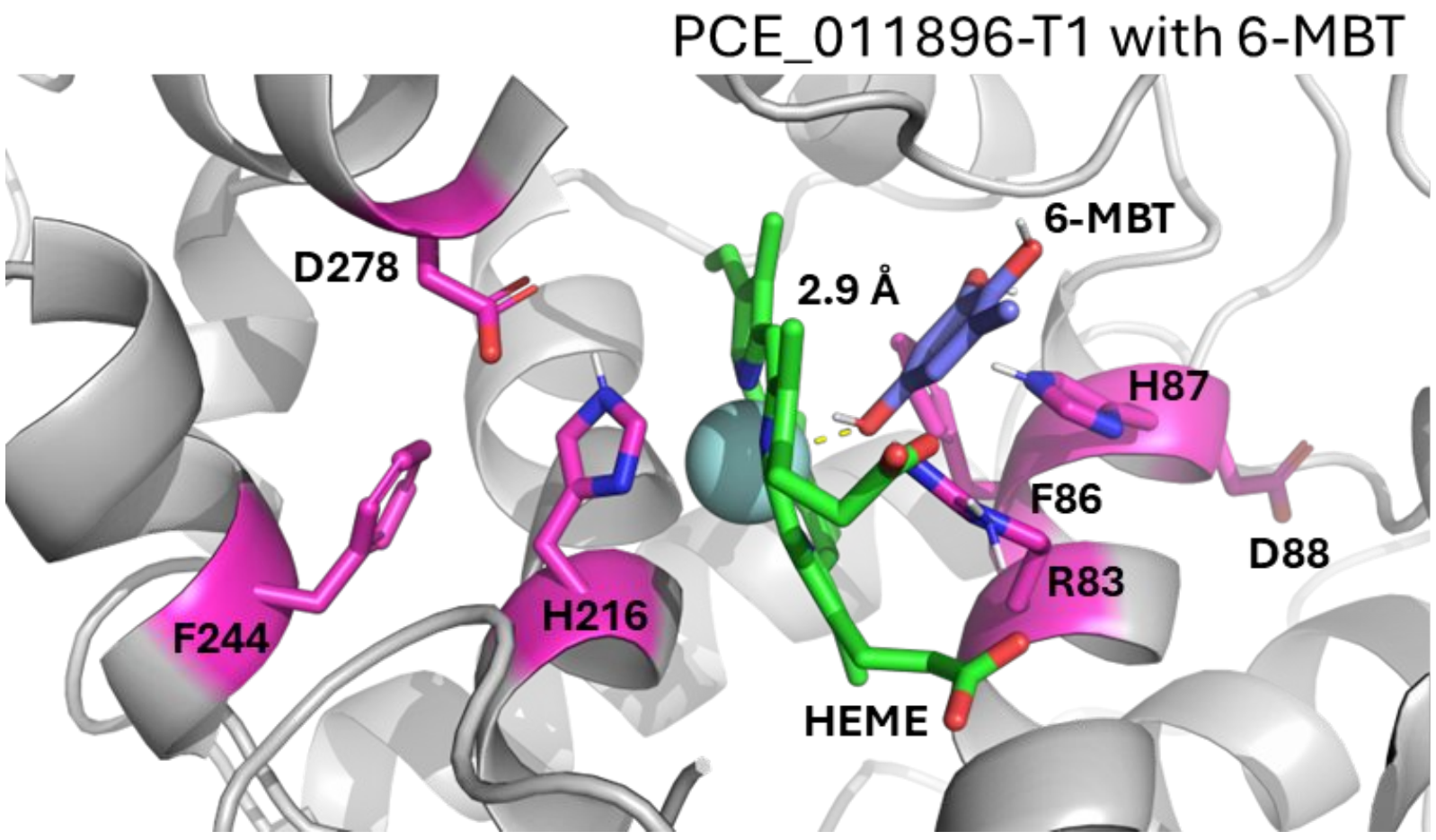

Figure S15: Visualization of the docking results of 6-methyl-1,2,4-benzenetriol (6-MBT, blue) and the cofactor heme (green, central Fe(III) ion light blue) for the putative heme peroxidase **PCE_011896-T1**. For the docking, the top-scoring pose was selected. Amino acid residues involved in ligand or substrate binding, as well as those with catalytic functions, are labeled and shown in magenta. The dashed yellow line marks the distance between the target atoms of cofactor and substrate.

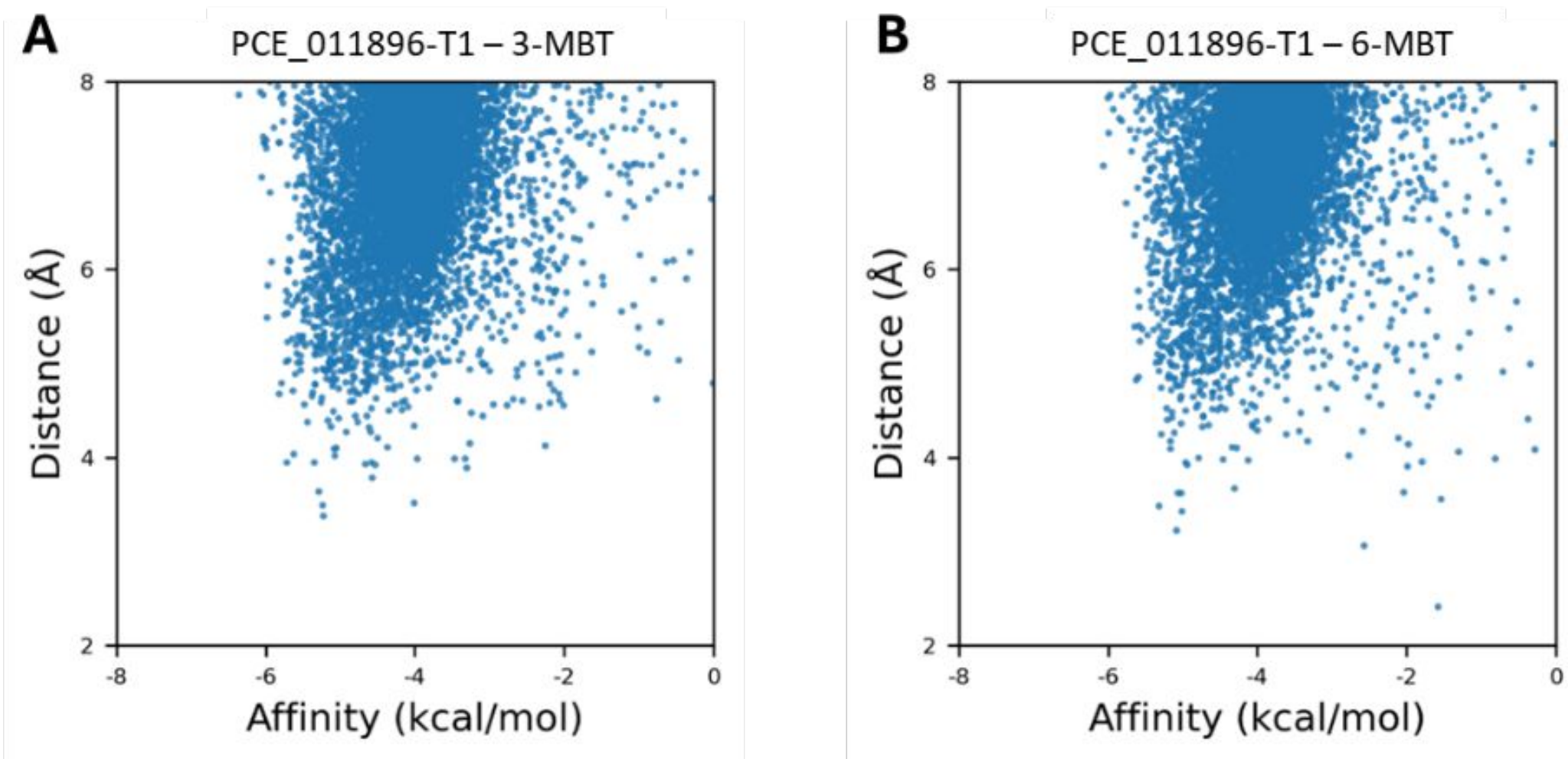

Figure S16: Distance/Affinity plots for the dockings of putative heme peroxidase **PCE_011896-T1** with the substrates (**A**) 3-MBT and (**B**) 6-MBT.
